# TGFβR Hyperactivation Drives CD103⁺/ITGB2⁺ TRM Cell Accumulation in High-Fat Diet-Exacerbated CIA

**DOI:** 10.7150/ijbs.124386

**Published:** 2026-03-30

**Authors:** Sijia Yan, Jielin Sun, Khan Zara Ahmad, Xuan Wang, Lei Tong, Qiangdongzi Mao, Qingwen Wang, Adeleh Divsalar, Edwin Cheung, Chaofu Wang, Xu Wang, Xianting Ding

**Affiliations:** 1 Department of Pathology, Ruijin Hospital and College of Basic Medical Sciences, Shanghai Jiao Tong University School of Medicine, Shanghai 200025, China.; 2 Key Laboratory of Cell Differentiation and Apoptosis of Chinese Ministry of Education, Shanghai Jiao Tong University School of Medicine, Shanghai 200025, China.; 3 Institute of Translational Medicine, Shanghai Jiao Tong University, Shanghai 200240, China.; 4 Department of Laboratory Medicine, Division of Pathology, Karolinska Institutet, Stockholm 17177, Sweden.; 5 Department of Anesthesiology and Surgical Intensive Care Unit, School of Medicine and School of Biomedical Engineering, Xinhua Hospital, Shanghai Jiao Tong University, 200240, Shanghai, China.; 6 Department of Cell and Molecular Sciences, Faculty of Biological Sciences, Kharazmi University, Tehran 15719-14911, Iran.; 7 Cancer Centre, Centre for Precision Medicine Research and Training, Faculty of Health Sciences, University of Macau, Taipa 999078, Macau SAR.

**Keywords:** obesity, rheumatoid arthritis, collagen-induced arthritis, TGFβR hyperactivation, tissue-resident memory T cells, asiaticoside

## Abstract

Obesity exacerbates rheumatoid arthritis (RA). However, the underlying mechanisms remain incompletely defined. Elucidating these mechanisms can help the identification of novel therapeutic targets. Herein, we used high-fat diet (HFD)-induced obese collagen-induced arthritis (CIA) mice to investigate these mechanisms. Immunohistochemistry revealed that obesity exacerbated joint inflammation and cartilage degradation. Next, integrated label-free quantitative proteomics and cytometry by time-of-flight (CyTOF) were used to characterize lymphocyte subsets. Proteomic profiling identified 26 differentially expressed proteins in obese versus lean CIA mice, including the transcription factors EOMES and KLF2, the TGFβ receptor (TGFβR) signaling component TGFBR2, and the tissue-resident memory (TRM) T cell marker CD103. CyTOF analysis revealed a robust 3.0-fold increase (*P =* 0.0043) in the proportion of CD103⁺ TRM cells among CD3⁺ T cells in obese CIA mice, characterized by a large effect size. Immunofluorescence results confirmed this increase in synovial tissues. Treatment with asiaticoside (a TGF-β/Smad-suppressing triterpenoid) significantly reduced TRM cell proportions (*P* < 0.05) and ameliorated symptoms in obese CIA mice. Collectively, these findings establish a novel mechanistic axis in which obesity-induced TGFβR-hyperactivation promotes TRM cell accumulation, which exacerbates arthritis severity in this RA model. Our findings provide a preclinical rationale for targeting TGFβR/TRM in human RA with obesity as a comorbidity.

## Introduction

Rheumatoid arthritis (RA) is mainly characterized by symptoms such as swelling, pain, and stiffness in the joints, most commonly the hands, wrists, and knees. Without timely intervention, this disease can lead to irreversible joint damage, deformity, and ultimately, disability 1. Consequently, individuals with RA have a higher risk of cardiovascular disease and a shorter lifespan than the general population 2, 3. Moreover, RA prevalence is age-related, being noticeably higher among older populations 4. According to the National Institutes of Health, approximately 1.3 million adults in the United States suffer from RA; globally, the prevalence is approximately 1% 5.

RA progression is influenced by both genetic and environmental factors. Obesity is a well-established contributing factor for RA development and poor outcomes. Indeed, a primary case-control study in England indicated that obesity (BMI ≥ 30 kg/m²) was associated with a substantially increased risk for developing RA (adjusted odds ratio = 3.74; 95% confidence interval: 1.14-12.27) 6. Furthermore, a retrospective longitudinal cohort study demonstrated that obesity predicted a poor treatment response in patients with RA 7. A meta-analysis corroborated this, finding that patients with obesity and RA were 40% less likely to achieve disease remission and only half as likely to sustain remission following pharmacotherapy 8.

Despite this strong epidemiological association, the precise molecular and cellular mechanisms through which obesity exacerbates RA remain incompletely elucidated. One potential mechanism involves the association between obesity and chronic inflammation. White adipose tissue acts as an active endocrine organ, secreting adipocytokines (e.g., leptin and adiponectin) and promoting the production of pro-inflammatory mediators, such as TNF-α and IL-6 9-11. These molecules exhibit immunomodulatory properties and affect catabolism, matrix degeneration, apoptosis, and inflammation in musculoskeletal cells 12-14. Key inflammatory mediators, including TNF-α, IL-1, and IL-6, play critical roles in RA pathogenesis and progression, potentially mediating processes such as T and B cell recruitment and activation, angiogenesis, chemotaxis, vascular permeability, and matrix metalloproteinase production 15. However, the association between specific adipocytokines and systemic inflammation in patients with RA and obesity appears complex and may vary among patients. For instance, in overweight/obese patients with RA, serum levels of IL-1β and IL-21 are positively correlated with BMI, while IL-6 and TNF-α levels show no such association. Serum IL-17A and GM-CSF exhibit weak positive correlations with BMI, whilst IFN-γ levels are negatively correlated 16.

Moreover, obesity exacerbates inflammatory arthritis in mice. Elevated levels of anti-type II collagen (CII) IgG and anti-CII IgG2a in sera, an increased number of Th17 cells in splenocytes, and heightened IL-17 expression in the spleen and joint synovium were observed in obese collagen-induced arthritis (CIA) mice compared with those in lean CIA mice. Jhun et al. demonstrated that obesity exacerbates joint inflammation through Th17 differentiation and IL-17 production in the synovium 17. Although Th17/IL-17 pathways provide a plausible link between obesity and RA, further research to elucidate the other intricate mechanisms underlying the effects of obesity on RA development is necessary.

The development of high-throughput technologies, such as next-generation sequencing and advanced mass spectrometry (MS), has revolutionized the discovery of key disease mechanisms. Modern label-free quantitative proteomics offers superior sensitivity and broader applicability for comprehensive proteomic analyses compared to labeled MS approaches. We leveraged these advantages to investigate the molecular mechanisms underlying obesity-exacerbated CIA using DBA/1 mice. These mice develop more severe CIA under high-fat diet (HFD) conditions compared to the commonly used C57BL/6 mice, providing a robust model to investigate the obesity-CIA relationship 18-20. Specifically, this study aimed to investigate how HFD-induced obesity exacerbates CIA and identify key biomarkers involved. These findings highlight therapeutic targets of human RA with comorbid obesity.

## Materials and Methods

### Animal models of obesity-exacerbated CIA

To mitigate interference from sex differences, 20 male DBA/1 mice (8-week-old, 15-17 g, and specific pathogen-free) were obtained from SLAC Laboratory Animal Co., Ltd. (Shanghai, China). Throughout the study, five mice were housed per cage with standard lab chow provided and water available ad libitum. After acclimation for one week under controlled conditions (22 ± 3 °C, 40-60% humidity, background noise of 40 ± 10 dB, and a 12:12 h light-dark cycle), the mice were randomly assigned to receive either a normal diet (ND, 10% kcal fat, SLAC) or an HFD (60% kcal fat, D12492, Research Diets) for 7 weeks, using a computer-generated random number sequence to ensure unbiased allocation. Following this dietary intervention, a second randomization procedure was performed within each diet group to assign mice to the final experimental cohorts: the established obese (HFD) mice were randomly distributed into obese CIA and obese Control groups, while the lean (ND) mice were allocated to lean CIA and lean control groups, ensuring baseline comparability for the subsequent CIA induction. The animal study design was approved by the Animal Research Ethics Committee of Shanghai Jiaotong University (Approval No.: 20220508), and all experiments were conducted in compliance with the ARRIVE 2.0 guidelines, the 3Rs principles (replacement, reduction, and refinement) for animal research, and the Regulations for the Administration of Laboratory Animals of the People's Republic of China 20.

### Induction of CIA

CIA was induced in DBA/1 mice following a validated protocol: chicken CII (Chondrex, Redmond, WA, USA) was dissolved to 2 mg/mL in 0.5 M acetic acid and incubated at 4 °C overnight^21^. Then, the solution was emulsified with an equal volume of Complete Freund's adjuvant (CFA; Sigma, Darmstadt, Germany) as described in reference 22. At 16 weeks of age, the mice were randomized and immunized. Mice in the CIA group (five lean CIA and five obese CIA mice) received intradermal injection of the CII/CFA emulsion. A booster immunization was administered two weeks later. Control mice (five lean control and five obese control mice) received CFA emulsion alone following the same schedule. One mouse in the lean CIA group died one week after the booster immunization, likely due to the severity of arthritis or complications from the immunization. The key proteomic findings were validated using cytometry by time-of-flight (CyTOF) in an expanded animal cohort (final lean CIA n = 6; all other groups, n = 5). This cohort incorporated two additional lean CIA mice, which were handled using the same protocol as the original animals. To ensure data comparability, we performed statistical analysis between the two independent cohorts of lean CIA mice (original n = 4 and new n = 2) and confirmed no significant differences in key disease parameters (P > 0.05), allowing for data pooling.

### Drugs and their administration

Asiaticoside (MedChemExpress, HY-N0436) was prepared as a 2.0 mg/mL solution in corn oil according to the manufacturer's instructions. This formulation was administered via daily oral gavage at a volume of 10 mL/kg body weight (up to 20 mg/kg/day), starting from the day of the second immunization and continuing for 21 days. The selected 20 mg/kg dose was well within the therapeutically effective range established in preclinical studies (typically 5-45 mg/kg), as supported by standard cross-species dose conversion principles 23. Most importantly, this specific dose had been validated in a mouse model, in which oral administration of 20 mg/kg asiaticoside was identified as the most effective dosage for exerting its biological activity 24. Control mice received an equivalent volume of the corn oil-based vehicle (prepared using the same protocol but without asiaticoside) following the same schedule.

### Patients and Samples

Synovial tissue samples were obtained from four patients diagnosed with rheumatoid arthritis at Renji Hospital. This study was conducted in accordance with the Declaration of Helsinki. This study involving human synovial tissue samples was reviewed and approved by the Ethics Committee of Renji Hospital South Campus, Shanghai Jiaotong University School of Medicine (Approval No.: RA-2020-506). All participants provided written informed consent. The samples were collected via ultrasound-guided puncture or arthroscopic synovial biopsy for subsequent pathological classification and molecular analysis.

### Histological and immunohistochemical analysis

Knee joints from mice in each group were fixed in 4% paraformaldehyde overnight, decalcified in 10% ethylenediaminetetraacetic acid (EDTA) solution for one week, embedded in paraffin, and sectioned into three μm-thick slices. For histological examination, sections were stained with Hematoxylin and Eosin (H&E) for general morphology and with Toluidine Blue for assessment of proteoglycan deposition (Servicebio, Wuhan, China). All stained sections were digitally scanned using a whole-slide scanner (LG-S80; Servicebio) for subsequent analysis.

Histopathological scoring was performed independently by two investigators blinded to the experimental groups, according to the criteria provided in the Supplementary Materials. The scoring results between the two investigators showed high consistency.

For immunohistochemical analysis, articular cartilage sections from all groups were processed as follows: deparaffinized in xylene, rehydrated through a graded ethanol series, and subjected to antigen retrieval using pepsin solution (Solarbio, Shanghai, China). Conventional immunohistochemistry (IHC) was performed using specific primary antibodies, followed by incubation with HRP-conjugated secondary antibodies and development with a DAB chromogenic system. Multiplex immunofluorescence (mIF) staining was performed on similarly pretreated sections using a TSA Plus Fluorescence Kit (Servicebio). Fluorescence images were acquired using a confocal microscope (TCS SP8; Leica Microsystems, Wetzlar, Germany) and quantitatively analyzed using ImageJ software (National Institutes of Health, USA).

### Pretreatment of lymphocyte samples for proteomic analysis

The lymph nodes of mice were processed into cell suspensions using cold phosphate-buffered saline in a 70 μm cell strainer and transferred to a 15 mL centrifuge tube. Mouse lymphocytes were lysed in SDT buffer (4% sodium dodecyl sulfate, 100 mM Tris-HCl, pH 7.6), followed by sonication and boiling for 15 min. Proteins were acetone-precipitated to remove SDS, and redissolved in 8 M urea/100 mM Tris-HCl (pH 8.5). After centrifugation at 14,000 × *g* at 4 °C for 10 min, we quantified the supernatant using the Bicinchoninic Acid Protein Assay Kit (Beyotime, Shanghai, China). Samples were prepared following protocols outlined in a previous study 25. Briefly, the cell precipitate was resuspended and dissolved in water and trifluoroethanol. In ammonium bicarbonate buffer, disulfide bonds were reduced with dithiothreitol and alkylated with iodoacetamide. After trypsin digestion, the peptide mixture was desalted and purified using a C18 cartridge (Sigma-Aldrich, Darmstadt, Germany).

### MS and data analysis

Lyophilized peptides were reconstituted in 40 μL of 0.1% formic acid and analyzed by liquid chromatography-tandem mass spectrometry (LC-MS/MS) using an EASY-nLC 1200 system (Thermo Fisher Scientific, Waltham, MA, USA) coupled to a TIMS-TOF Pro mass spectrometer (Bruker Daltonics, Bremen, Germany) equipped with a CaptiveSpray ion source. Buffer A contained 0.1% formic acid in water, whereas buffer B contained 0.1% formic acid in acetonitrile (100% acetonitrile). Initially, the column was equilibrated with 100% buffer A before the sample was loaded onto an analytical column (25 cm × 75 μm, C18 packing 1.6 μm, IonOpticks, Collingwood VIC Australia). The separation was performed at a flow rate of 300 nL/min.

The MS data were analyzed using the MaxQuant software version 1.6.14.0 and searched against the UniProt_Musculus_17056_20210125 database. A label-free quantification algorithm was used for the quantitative analysis 26. For each biological sample, a single LC-MS/MS run was performed (i.e., no technical replicates). Protein quantification was based on the MaxLFQ algorithm integrated in MaxQuant, which utilizes the extracted ion currents of peptides and corrects for between-run variations 27. Prior to statistical testing, the LFQ intensity data were log-transformed and normalized using the MaxQuant's built-in “delayed normalization” function, which applies a width adjustment of the data distribution to correct for systematic errors 28. MS data were acquired in data-dependent acquisition (DDA) mode, and MaxQuant effectively identified peptide features in the raw data, using graphical integration to calculate intensity values. Following the acquisition of raw files of the mass spectra, we set the grouping, database, and post-translational modifications. MaxQuant automatically conducted database matching and extracted intensity data for label-free quantification and qualitative protein results. In the qualitative analyses, the algorithm used the false discovery rate (FDR) principle for data screening, with both peptide and protein FDRs maintained at ≤ 0.01. For pairwise group comparisons (obese CIA vs lean CIA), proteins with fold changes ≥ 1.5 and *P* < 0.05 (Student's t-test) were considered differentially expressed proteins (DEPs).

### Bioinformatics analysis

All protein sequences were aligned against the database obtained from NCBI (ncbi-blast-2.2.28+-win32.exe), and only sequences within the top 10 that exhibited an E-value ≤ 1e-3 were retained. The Gene Ontology (GO) terms (database version: go_201504.obo) corresponding to the sequence with the highest Bit-Score were identified via Blast2GO and InterProScan predictions 29. Only terms supported by both tools were retained. After mapping, these GO terms were annotated on the target protein sequence, considering both the similarity between the target protein and the aligned sequence, as well as the reliability of the GO term sources. Pathway analysis was conducted using the Kyoto Encyclopedia of Genes and Genomes (KEGG) database. Fisher's exact test was used to enrich GO or KEGG terms by comparing the ratio of DEPs to total proteins associated with the GO or KEGG terms. R (version 4.4.2) was used to generate heatmaps of the quantitative data of the target protein set after normalization.

### Pretreatment of lymphocyte samples for CyTOF

The cell suspension of mouse lymphocytes was prepared as described previously. Subsequently, preheated serum-free Dulbecco's Modified Eagle Medium (DMEM; Servicebio) was added, and the suspension was centrifuged at 300 × *g* for 5 min at 25 °C. The supernatant was carefully removed using pipettes, and the cell pellet was resuspended in 1 mL of preheated DMEM containing 10% fetal bovine serum. Then, the cells were incubated for 30 min before adding 50 ng/mL Phorbol 12-myristate 13-acetate and 500 ng/mL ionomycin (both from Sigma-Aldrich) to each 1 mL of cell suspension, and the mixture was cultured at 37 °C for 4 h. After washing thrice with serum-free DMEM, the cells were stained with cisplatin for 5 min at 37 °C to differentiate viable and non-viable cells. Staining was terminated by adding cell-staining buffer (Fluidigm, San Francisco, CA, USA) and centrifugation. The supernatants were immediately discarded, and the cells were fixed in a 1.6% paraformaldehyde solution at 25 °C for 10 min. Following additional washes, the cells were cryopreserved in DMSO-containing cell staining buffer (CSB) at -80 °C.

### Antibody staining and data acquisition using CyTOF

Cryopreserved cells were thawed for 5 min in a pre-warmed (37 °C) water bath, and 3 × 10^6^ cells were transferred into CSB for antibody staining, as previously described 30. After surface staining, cells were fixed with 1.6% PFA for 10 min at room temperature (20-25°C) and permeabilized with 90% ice-cold methanol (30 min, 4 °C). Then, cells were rinsed twice with 2 mL of CSB and incubated overnight at 4 °C with 1 mL of DNA Intercalator (Fluidigm), then diluted in Fix and Perm Buffer (Fluidigm) to achieve a final concentration of 125 nM. This step facilitates the differentiation of singlets, doublets, and triplets. Before data acquisition, the cell pellets were resuspended in distilled water containing 10% EQ Four Element Calibration Beads (Fluidigm) and filtered through a 35 µm membrane. The cells were analyzed using the Helios system (Fluidigm) across multiple runs at an acquisition rate of approximately 500 events per second, adhering to the manufacturer's default settings 31.

### Data processing and statistical analysis

Helios short-term signal fluctuations were standardized using EQ bead signals before data export and analysis 32. Subsequently, to enable cross-sample comparison, the expression level of each surface marker was normalized within each sample by dividing the raw signal intensity by the 99.5th percentile value of that marker's distribution in that sample, yielding a unitless normalized expression value 33. Then, recorded events were gated based on DNA content and cell length to exclude fragments and dimers. The Cytobank platform (www.cytobank.org) was used for the spanning-tree progression analysis of gating; variational inference for stochastic neighbor embedding (viSNE) graph and density-normalized event (SPADE) analyses were performed as previously described 34. The markers used for the viSNE analyses were CD4, CD8a, Eomes, CD69, CD103, T-BET, CTLA4, PD1, ITGB2, FOXP3, CD45, CD3e, CD44, CD62L, RORγt, and KLF2. A series of gating of T cell subpopulations is shown in Fig. S8. Major cell subpopulations were annotated in the viSNE based on the expression of established markers of various cell types. FlowSOM clustering was performed using the Cytofkit R package to analyze a combined sample of 150,000 cells (secondary sampling of 10,000 cells per sample) using all markers 35. The sample size of 10,000 cells per file was determined based on common practices in high-dimensional cytometry, which suggest that this number is sufficient to capture even rare cell populations present at frequencies as low as 0.5-1% while maintaining computational efficiency for clustering algorithms 33.

All analyses were performed using GraphPad Prism 9 (GraphPad Software, Inc., San Diego, CA, USA). Data are presented as the mean ± SD. For the key two-group comparisons (e.g., obese CIA vs. lean CIA), the Mann-Whitney U-test, which is robust for small sample sizes, was employed. To quantitatively assess the robustness of these findings, a post-hoc power analysis was conducted using the GPower software (version 3.1.9.7, Universität Düsseldorf, Düsseldorf, Germany), which confirmed that the achieved statistical power for this comparison far exceeded the conventional threshold of 80% 36. For comparisons across multiple groups, two-way ANOVA (factors: diet and immunization) with Tukey's post-hoc test was used. For correlation analyses (e.g., TRM subset frequencies versus arthritis pathology scores), Spearman correlation coefficients were calculated. Given the exploratory nature of these correlation analyses and to control the FDR across multiple tested correlations, *P*-values were adjusted using the Benjamini-Hochberg procedure 37. In all cases, differences were considered statistically significant at *P* < 0.05.

## Results

### Impact of obesity on arthritis severity in CIA mice

**Figure 1:**
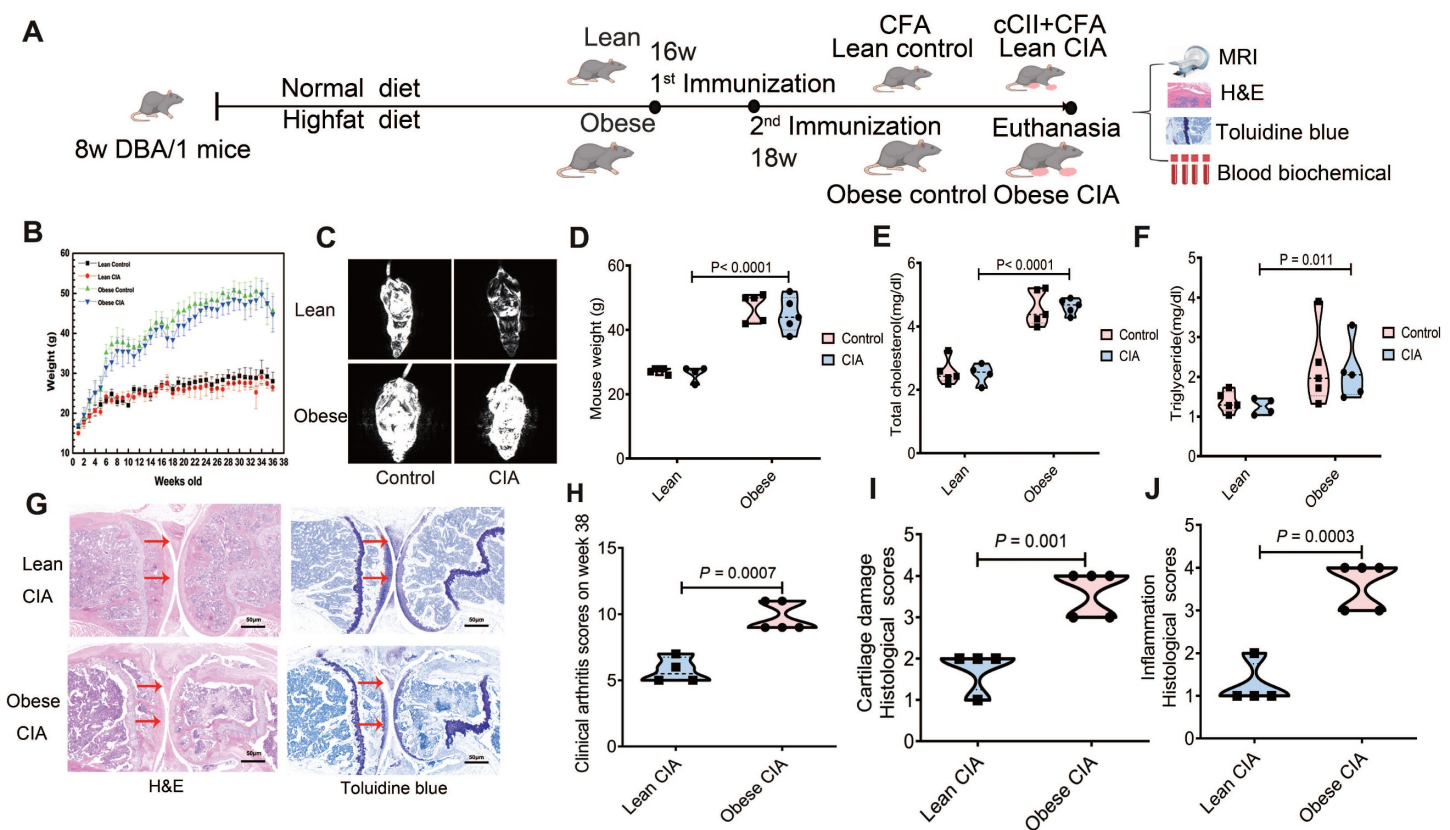
** Obesity exacerbates CIA in DBA/1 model mice.** (A) Schematic representation of the diet groups, timeline of immunization, and experimental workflow. (B) Body weight changes from week 0 to the endpoint. n = 5/group initially; one lean CIA mouse died at week 3 (excluded). Final n: obese control = 5, lean control = 5, obese CIA = 5, lean CIA = 4. Data represent the mean ± SD). (C) MRI results depict the fat distribution across different groups of mice. Visceral fat mass significantly increased in obese groups. (D-F) Metabolic parameters measured at the endpoint for each experimental group (lean control, n = 5; lean CIA, n = 4; obese control, n = 5; obese CIA, n = 5). (D) Body weight, (E) Serum triglyceride content, and (F) Total cholesterol levels. Data are presented as the mean ± SD. Statistical significance was determined using the Mann-Whitney U test, focusing on the main effect of the diets. *P* < 0.05 indicates a significant effect of a high-fat diet (HFD) compared to a normal diet (ND). No significant interaction between diet and CIA induction was observed for these parameters. (G) Representative images of H&E and toluidine blue staining in knee joint sections of lean and obese CIA mice. Arrows indicate lesions and abnormalities in the joints. Scale bars: 50 μm. (H) CIA clinical scores were evaluated on a 0-4 scale per paw (scoring criteria in the Supplementary Material), and were significantly higher in obese CIA mice (n = 5) vs. lean CIA mice (n = 4; Mann-Whitney U test, *P* < 0.05). (I-J) Histological scores for cartilage damage and inflammation in the paws of obese CIA mice (n = 5) and lean CIA mice (n = 4, Mann-Whitney U test, *P* < 0.05). Each dot represents one mouse.

To investigate the impact of obesity on CIA, twenty DBA/1 mice at 8 weeks old were initially randomly selected to receive either ND or HFD for a 7-week dietary intervention, following a one-week acclimation period (Fig. 1A). The obese mice exhibited significantly higher body weights than the lean mice at 16 weeks of age (23.51 ± 1.28 g vs. 37.82 ± 1.75 g, *P* < 0.0001). Subsequently, a second randomization round was performed within each diet group to assign mice with comparable baseline body weights within the lean and obese cohorts, confirmed prior to immunization, to the CIA induction or control groups (obese control vs obese CIA: 36.41 ± 2.58 g vs. 39.20 ± 2.14 g, *P* > 0.05; lean control vs. lean CIA: 23.81 ± 1.47 g vs. 23.21 ± 0.98 g, *P* > 0.05; Fig. S1A-B).

Weekly body weight monitoring demonstrated that obese mice maintained significantly higher body weights than lean mice throughout the study (Fig. 1B). To assess body composition, whole-body magnetic resonance imaging (MRI) was performed prior to sacrifice, which revealed pronounced adiposity in obese mice (Fig. 1C). The mean body weights at this stage were 45.78 ± 2.54 g in obese mice versus 27.01 ± 1.38 g in lean mice. To confirm the metabolic impact of the HFD intervention, we compared body weight and serum lipid profiles across all experimental groups using two-way ANOVA. This analysis revealed a significant main effect of the diets, as HFD-fed mice (obese control: n = 5; obese CIA mice: n = 5) exhibited markedly increased body weights, serum triglyceride contents, and total cholesterol levels compared to their ND-fed counterparts (lean control: n = 5; lean CIA: n = 4) (Fig. 1D-F). No significant main effect of CIA induction or diet-by-CIA interaction was observed for these parameters (all *P* > 0.05), indicating that the metabolic dysregulation was primarily driven by the dietary intervention itself.

CIA development was monitored, and arthritis severity was scored. The scoring system, timeline scores, and individual mouse disease severity are detailed in Tables S1 and S2. Both lean and obese CIA mice developed typical arthritis symptoms, with visible swelling in the paws (Fig. S1A-D). Notably, obese CIA mice displayed more pronounced joint enlargement (Fig. S1C-D) compared to the predominantly localized digit swelling observed in lean CIA mice. After euthanasia, knee joints were harvested and processed for histological analysis. Tissues were decalcified and stained with H&E as well as toluidine blue (Fig. 1G). Synovial inflammation and cartilage damage were assessed using detailed histological scoring systems (Tables S3 and S4).

Obese CIA mice exhibited significantly higher clinical arthritis scores than lean CIA mice (*P* = 0.0007; Fig. 1H). Histological analysis revealed exacerbated pathology in obese mice. Synovial inflammation scores were significantly higher (*P* = 0.001), characterized by increased synovial edema, hyperplasia, inflammatory cell infiltration, and pannus formation. Articular cartilage destruction was also significantly more severe in obese CIA mice (*P* = 0.0008; Fig. 1I and J). Collectively, these findings demonstrate that obesity significantly exacerbated the severity of CIA.

### Label-free quantitative proteomic analysis

**Figure 2:**
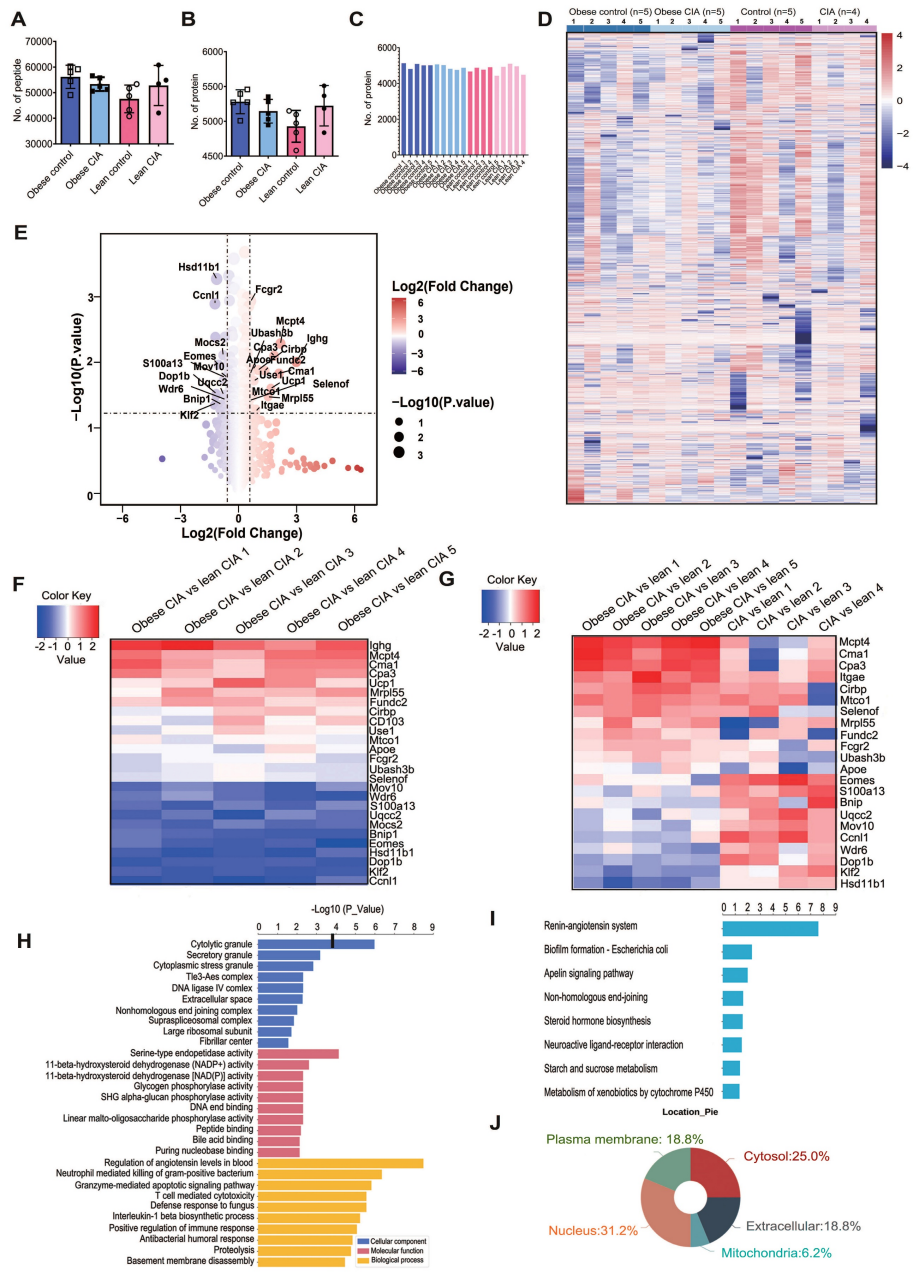
** Proteomic profiling of lymphocyte samples showing the DEPs between the diet groups.** (A-B) Distribution of (A) quantified peptides and (B) proteins across groups (Obese control: n = 5; Obese CIA: n = 5; Lean control: n = 5; Lean CIA: n = 4). Error bars = SD. (C) Distribution of proteins in each lymphocyte sample. (D) Heatmap of the final 4775 reserved proteins (Supplemental Tables S7). (E) The volcano plot of these DEPs. (F-G) Heatmaps of log₂ fold-change for 26 DEPs in (F) obese CIA vs lean CIA; (G) obese CIA vs obese control. (H) GO enrichment of DEPs (obese CIA vs lean CIA; FC≥1.5, *P* < 0.05). Top terms based on -log₁₀(P-values) are shown (hypergeometric test; *P* < 0.001). (I) KEGG pathway enrichment of DEPs (sorting and statistics as in E). (J) Subcellular localization analysis of DEPs.

To elucidate molecular mechanisms underlying obesity-exacerbated arthritis following systemic antigen challenge, we performed label-free quantitative proteomic analysis on lymphocytes isolated from popliteal lymph nodes of mice from four experimental groups: lean control (ND), lean CIA (ND+CII), obese control (HFD), and obese CIA (HFD+CII). LC-MS/MS analysis of protein digests identified 73,048 unique peptides (Table S5), with group-specific peptide counts ranging from 47,568 to 56,203 (Fig. 2A). Database search results (using Andromeda) indicated high confidence in the peptide identification results, with 99.7% (72,855) of the identified peptides achieving an FDR of < 1%. Approximately 74.2% of the peptides scored > 60 (Andromeda Scoring Criteria), indicating high confidence. The average spectral count per peptide was 101.1 (Fig. S2A-D).

Among the identified peptides, 6,367 proteins were detected (Table S6), with an average of 4,928-5,281 proteins per group (Fig. 2B). Critically, 83.6% of proteins (5,323/6,367) were represented by > 2 unique peptides (mean 12.97 unique peptides/protein), while 527 and 494 proteins were represented by exactly two and one unique peptide(s), respectively. The high peptide coverage indicates accurate protein identification. The distribution of proteins across samples is shown in Fig. 2C.

To ensure robust quantitative analysis, we selected proteins identified by ≥ 2 unique peptides and detected in ≥ 3 biological replicates per experimental group. This resulted in 4,775 high-confidence proteins (Table S7). Hierarchical clustering of these proteins revealed distinct expression patterns across groups (Fig. 2D), demonstrating significant proteomic alterations associated with both diet (HFD vs ND) and disease state (CIA vs control), which potentially indicate key drivers of obesity-exacerbated arthritis.

### Proteomic alterations in obesity-exacerbated CIA

Proteins with a ≥ 1.50-fold and statistically significant (*P* < 0.05) change in expression were defined as DEPs. We identified 26 DEPs between obese and lean CIA mice: 15 were upregulated and 11 were downregulated in obese CIA mice (Table 1), collectively indicating profound dysregulation of immune and metabolic pathways. The global expression pattern of these DEPs, as shown in the volcano plot (Fig. 2E), highlighted several proteins of interest that were selected for further functional analysis.

Notably, obese CIA mice exhibited upregulation of the expression of immune-related proteins (IGHG, MCPT4, CMA1, and CPA3), the thermogenic marker UCP1, and APOE, a key regulator of lipid metabolism which is implicated in inflammatory arthritis-associated cartilage damage. Mast cell (MC) proteases (MCPT4, CMA1, and CPA3) were significantly upregulated, indicating enhanced inflammatory responses. UCP1 upregulation may indicate enhanced thermogenesis, potentially reflecting an adaptive metabolic response. Downregulation of RNA processing regulators (CCNL1) and transcriptional regulators (KLF2 and EOMES), as well as metabolic enzymes (DOP1B and HSD11B1), was observed.

Additionally, we identified 14 proteins uniquely expressed in obese CIA mice (ZFP809, JCHAIN, IGHA, KLK1, LAMA4, SIGLEC10, EPC2, STAB2, FYTTD1, KCTD10, FKBP11, TMEM263, HEMGN, and TESC), including immune regulators (IGHA and SIGLEC10) and extracellular matrix components (LAMA4). This suggests unique molecular features driving severe arthritis in the obese state. In contrast, only two proteins (ACCS and DDX11) were exclusively detected in lean CIA mice.

To explore DEP expression dynamics across metabolic and disease states, hierarchical clustering was performed on the 26 DEPs in obese CIA, lean CIA, and lean control mice (Fig. 2F). Clustering revealed that IGHG, UCP1, USE1, and MOCS2 were of low abundance in lean controls but were upregulated in both CIA groups. In contrast, the remaining 22 DEPs showed significant differential abundance between obese CIA and lean CIA mice (Fig. 2G), highlighting their potential role in obesity-driven arthritis exacerbation.

### Functional annotation of DEPs

Additionally, we conducted GO and KEGG enrichment analyses on DEPs 38, 39. GO enrichment analysis of DEPs categorized functions into Biological Processes (BP), Cellular Components (CC), and Molecular Functions (MF) (Fig. 2H). Among the enriched GO terms, BP showed the highest number of significant associations. Key enriched BP terms included regulation of systemic arterial blood pressure, neutrophil-mediated killing of gram-positive bacteria, granzyme-mediated apoptotic signaling pathway, and T cell-mediated cytotoxicity. KEGG pathway analysis identified the renin-angiotensin system (RAS) as the most significantly enriched pathway (FDR < 0.005) (Fig. 2I). This aligns with reports linking activation of RAS and pro-inflammatory cytokines (e.g., IL-1β) to increased RA severity 40, suggesting RAS may be a key mechanistic node in obesity-aggravated arthritis. Subcellular localization analysis revealed a significant enrichment of nucleus-localized proteins among DEPs (Fig. 2J), suggesting potential dysregulation of nuclear processes, such as transcriptional regulation, in obesity-exacerbated CIA.

### Protein expression changes in the lymphocytes of obese CIA mice

Compared with lean CIA mice, the expression of three MC markers, CMA1, MCPT4 and CPA3, was significantly increased in obese mice, indicating an increased presence of MCs within the lymphocyte population. Additionally, FcγRII (an inhibitory receptor expressed on B cells and myeloid cells) showed significantly higher expression in obese CIA mice, as depicted in Fig. S3A-D. Activated MCs can promote Th17 cell differentiation through cytokine secretion (e.g., IL-1β), especially in the memory CD4^+^ T cell population 41. However, as MC marker levels were also elevated in non-arthritic obese mice, MC accumulation alone is insufficient to explain arthritis exacerbation; instead, it may prime the inflammatory microenvironment for enhanced disease severity in the context of CIA.

**Figure 3:**
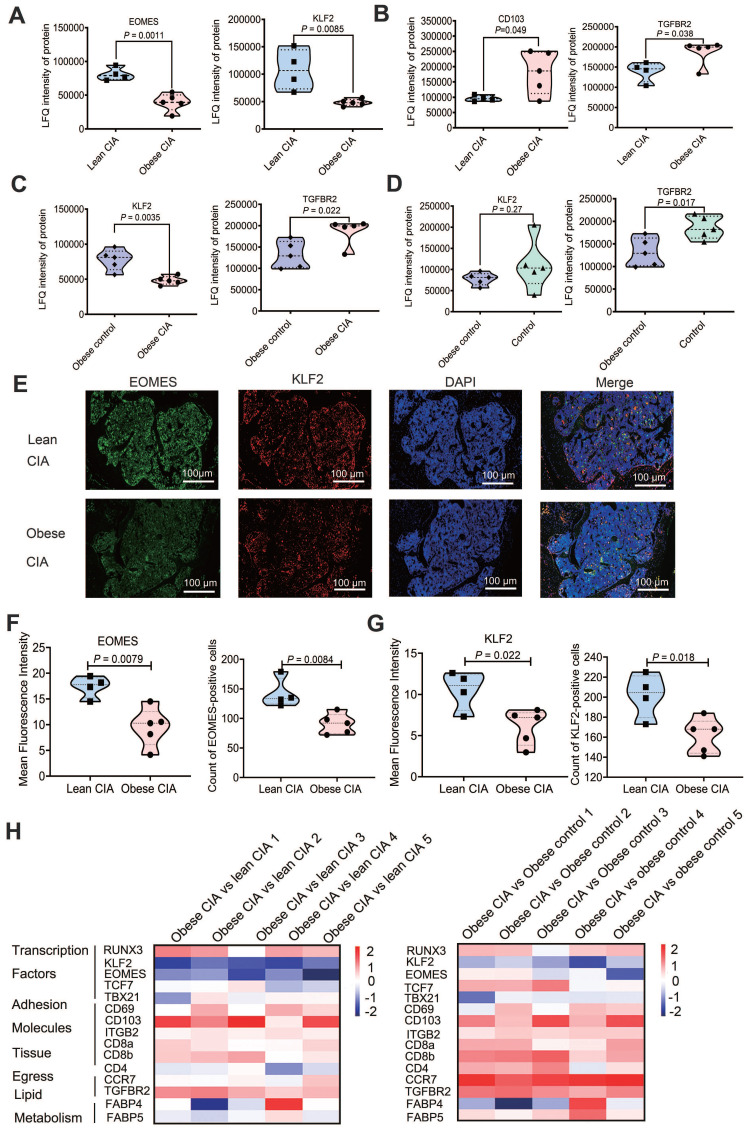
** Obesity dysregulates TRM-associated protein expression in CIA mice.** (A-B) Proteomic analysis of lymphocytes revealed significant differences in the expression of (A) transcription factors (EOMES and KLF2) and (B) TRM markers (CD103), and TGFβRII (TGFBR2) between obese and lean CIA mice (obese CIA: n = 5; lean CIA: n = 4; analyzed using the Mann-Whitney U test). (C-D) Differences in KLF2 and TGFβRII (TGFBR2) between obese control and CIA mice or obese control and control mice (Obese CIA: n = 5; Obese control: n = 5, Control: n = 5; determined using the Mann-Whitney U test). (E) Representative immunofluorescence images of bone marrow sections co-stained for KLF2 (red), EOMES (green), and DAPI (blue) in lean and obese CIA mice. Scale bars: 50 μm. (F-G) Quantification of nuclear EOMES and KLF2 mean fluorescence intensity (MFI) from immunofluorescence staining. MFI was quantified using the ImageJ software (Obese CIA: n = 5; Lean CIA: n = 4, Mann-Whitney U test). (H) Heatmap of TRM marker (CD69, CD103, and ITGB2) expression from proteomic data: Left: obese CIA vs lean CIA; Right: obese CIA vs obese control. Color scale: log₂ fold-change. Each dot represents one mouse.

Moreover, obese CIA mice displayed marked downregulation of the transcription factors EOMES (0.49-fold, *P* = 0.011) and KLF2 (0.44-fold, *P* = 0.042) in lymphocytes (Fig. 3A). KLF2 deficiency correlates with increased MMP9 expression and osteoclast activation, while EOMES reduction in T cell subsets is associated with impaired CD8⁺ T cell cytotoxicity and may indirectly promote Th17 responses 42-46. Additionally, CD103 and transforming growth factor-β receptor II (TGFβRII) expression levels were significantly higher in obese CIA mice than in lean CIA mice (Fig. 3B). Notably, this downregulation was specific to mice with CIA and obesity, as KLF2 expression was suppressed more in obese CIA mice compared with that in the obese control mice (Fig. 3C), indicating a synergistic effect of obesity and autoimmune challenge. Intriguingly, the upregulation of TGFβRII expression was opposite to its expression pattern in non-arthritic obese control mice (Fig. 3D).

Immunofluorescence and subsequent quantification of mean fluorescence intensity (MFI) using ImageJ (National Institutes of Health) revealed a significant reduction in nuclear EOMES and KLF2 staining in the bone marrow (Fig. 3E-G; EOMES MFI decreased by 45.1%, *P* < 0.01; KLF2 MFI decreased by 42.2%, *P* < 0.05). This aligns with previous reports of KLF2-mediated suppression of osteoclast genesis, highlighting synergistic dysregulation of transcriptional networks by obesity and arthritis, which may disrupt immune-metabolic homeostasis 22-26. These findings collectively suggest that the protein expression profile supports potential expansion of tissue-resident memory T (TRM)-like cells 47. Moreover, we identified 15 TRM-associated proteins that were differentially expressed in obese CIA mice compared to both lean CIA and obese control groups (Fig. 3H), further supporting TRM involvement and indicating obesity-driven TRM alterations in the arthritic context.

### Obesity enhances TRM cell subset abundance in CIA

**Figure 4:**
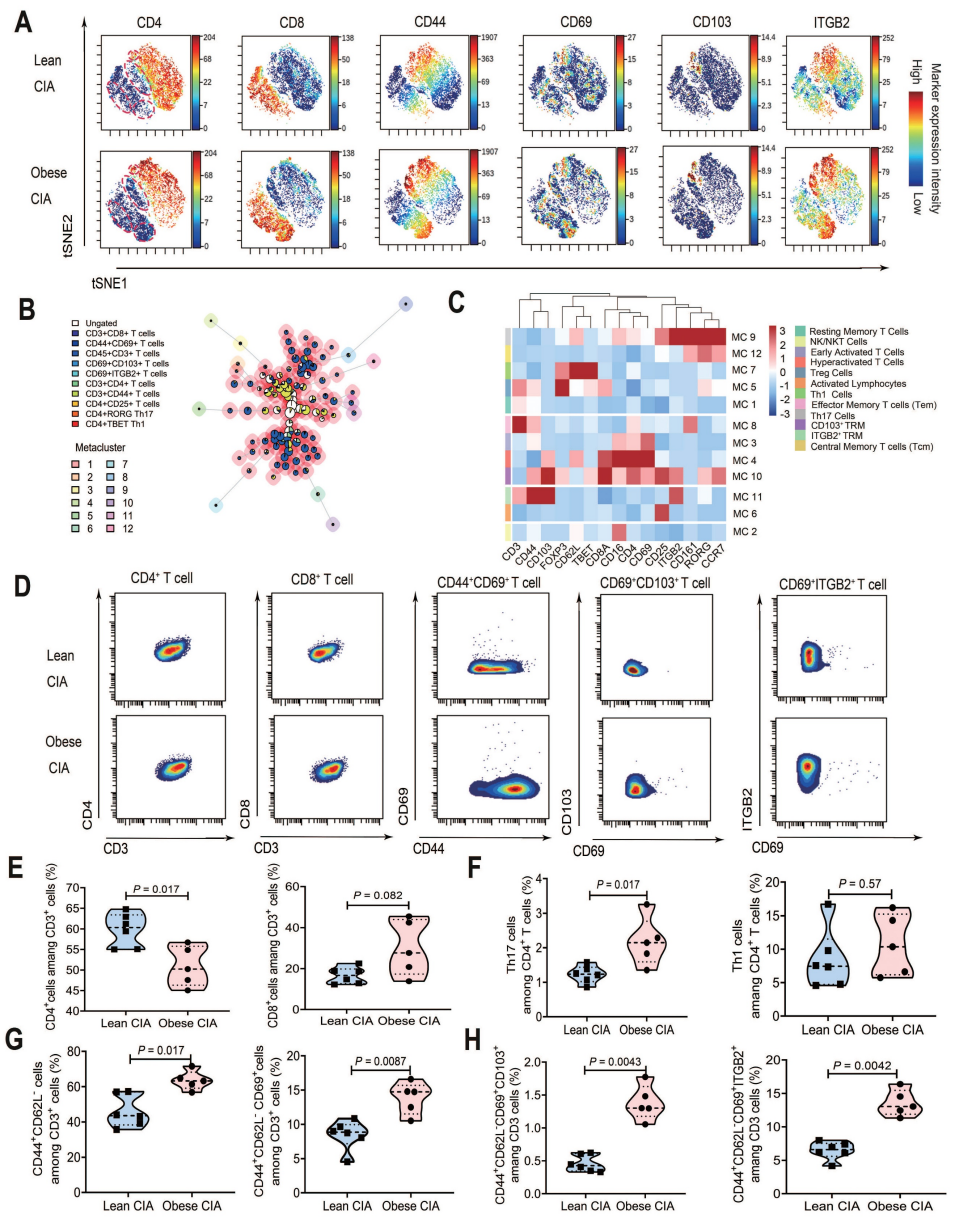
** Obesity altered T cell subset composition in CIA mice.** (A) viSNE analysis of T cells from lymphocytes (lean CIA vs obese CIA) showing the expression levels of 20 protein markers (e.g., CD4, CD103, and EOMES). (B) UMAP projection of FlowSOM metaclustering for CD45⁺ live cells, displaying 12 distinct metaclusters (MC1-MC12). (C) Heatmap of cluster-defining marker expression. Expression levels (z-score normalized) of key surface markers and transcription factors in each metacluster are shown. (D) Paired vertical bivariate plots comparing the abundances of CD4⁺ T cells, CD8⁺ T cells, and CD103⁺CD69⁺ TRM cells between lean and obese CIA mice. (E) Proportions of CD4⁺ and CD8⁺ T cells within CD3⁺ T cells (obese CIA: n = 5; lean CIA: n = 6; analyzed using the Mann-Whitney U test). (F) Proportions of Th1 (T-bet⁺) and Th17 (RORγt⁺) cells among CD4⁺ T cells (obese CIA: n = 5; lean CIA: n = 6; determined using the Mann-Whitney U test). (G) Proportion of naïve T cells (Tn: CD44⁻CD62L⁺) and TRM cells (CD44⁺CD62L⁻CD69⁺) within CD3^+^ T cells (obese CIA: n = 5; lean CIA: n = 6; analyzed using Mann-Whitney U test). (H) Proportion of TRM subpopulations (CD103⁺ and ITGB2⁺) within CD3⁺ T cells, defined by CD44⁺CD62L⁻CD69⁺ co-expression (obese CIA: n = 5; lean CIA: n = 6; analyzed using the Mann-Whitney U test). Each dot represents one mouse. Data for the lean CIA group were collected from two independent experimental cohorts (n = 4 and n = 2) to increase the sample size.

To explore the mechanistic link between obesity and RA exacerbation via T cell subsets, we performed high-dimensional CyTOF analysis of lymphocytes from obese CIA mice (n = 5) and lean CIA mice (n = 6; including two mice from a supplementary validation cohort). Statistical comparison confirmed that the key T-cell subset frequencies did not differ significantly between the two cohorts (P > 0.05 for all comparisons), justifying the pooling of data for subsequent analyses. Dimensionality reduction and visualization of the high-dimensional data were achieved using viSNE based on key surface markers (CD3, CD4, CD8, CD44, CD62L, CD103, CD69, and ITGB2), illustrating the continuum of phenotypic states and marker expression intensities (Fig. 4A).

FlowSOM unsupervised clustering of CD45⁺ living cells, incorporating lineage-defining transcription factors (T-bet, RORγt, and FOXP3), revealed 12 distinct metaclusters (Fig. 4B), which were annotated based on their unique marker expression profiles (Fig. 4C and Table 2). The analysis identified two TRM-associated populations: a canonical CD103⁺ TRM subset (MC10) exhibiting high expression of CD103 and CD69, and an ITGB2⁺ resident memory-like T cell population (MC11) characterized by elevated CD44 and ITGB2 but lacking CD103. Notably, the clustering resolved key functional T cell subsets including RORγt⁺ Th17 cells (MC9), T-bet⁺ Th1 cells (MC7), and FOXP3⁺ regulatory T cells (MC5).

To directly compare the abundance of key T-cell subsets, they were presented in bivariate plots in a paired vertical layout, displaying each population in the lean and obese CIA groups (CD4⁺, CD8⁺, CD44^+^CD69^+^ T Cells and TRM phenotypes) (Fig. 4D). The staining panel is detailed in Table S8. In obese CIA mice, the percentage of CD4⁺ T cells within CD3⁺ T cells was significantly reduced (*P* = 0.017), while that of CD8⁺ T cells showed a non-significant increase (Fig. 4E). Notably, the proportion of RORγt⁺ Th17 cells within CD4⁺ T cells was significantly higher in obese mice (*P* = 0.023), whereas T-bet⁺ Th1 cell abundance showed no significant change (Fig. 4F). Additionally, CD44⁺CD62L⁻ memory T (*P* = 0.017) and CD44⁺CD62L⁻CD69⁺ TRM cells (*P* = 0.0087) significantly increased in obese CIA mice (Fig. 4G).

Based on CD103 and ITGB2 expression, TRM cells were categorized into CD103⁺ TRM and ITGB2⁺ TRM (CD103⁻). The overall TRM pool (CD44⁺CD62L⁻CD69⁺) showed a 3.0-fold increase in CD103⁺ TRM (*P* = 0.0043) and 2.0-fold increase in ITGB2⁺ TRM (*P* = 0.0042) in obese CIA mice (Fig. 4H). Notably, this key finding regarding CD103⁺ TRM cells was supported by an exceptionally large effect size (Cohen's d = 3.75) and a post-hoc statistical power of 99.98%, confirming the robustness of this difference despite the modest sample size (obese CIA, n = 5; lean CIA, n = 6). Spearman's correlation analysis demonstrated strong positive associations between TRM cell subsets and both key pathological parameters of CIA. The frequency of CD103⁺ TRM cells was strongly correlated with inflammation scores (r = 0.94, *P* < 0.0001) and cartilage damage scores (r = 0.89, *P* = 0.0012). Similarly, the frequency of ITGB2⁺ TRM cells showed significant positive correlations with inflammation scores (r = 0.78, *P* = 0.012) and cartilage damage scores (r = 0.82, *P* = 0.0065).

This obesity-driven increase of TRM cell abundances across both CD4⁺ and CD8⁺ lineages reveals a novel mechanism for enhanced local immune activation in obese CIA. Strikingly, both CD4⁺ and CD8⁺ T cells exhibited expansions (CD103⁺ TRM: CD4⁺ *P* = 0.0043, CD8⁺ *P* = 0.023; ITGB2⁺ TRM: CD4⁺ *P* = 0.017, CD8⁺ *P* = 1.2×10⁻⁴) (Fig. S4A-B). Given the pathogenic potential of TRM subsets, we further analyzed PD1-associated TRM subtypes. Hobit-enriched CD103^high^PD1^low^ and granzyme K-enriched CD103^low^PD1^high^ TRM subsets were significantly increased in obese CIA mice across both CD4⁺ and CD8⁺ T cells (Fig. S4C-D), suggesting enhanced cytotoxic and residency programs.

### Obesity modulates canonical TGF-β pathway activity

To biochemically validate TGF-β pathway activation, we analyzed phosphorylation of the downstream effectors Smad2/3. Western blotting revealed significantly increased phospho-Smad2/3 (p-Smad2/3) levels in obese CIA mice than in lean CIA mice, with no concomitant change in total Smad2/3 protein levels (Fig. S5A-B), confirming obesity-induced hyperactivation of the canonical TGF-β/Smad pathway.

To assess pathway activity at single-cell resolution, we quantified surface expression of latency-associated peptide (LAP), a marker of active TGF-β signaling, using our CyTOF data. LAP expression was significantly upregulated in CD44⁺CD69⁺ T cells, ITGB2⁺ TRM cells, and CD103⁺ TRM cells (particularly the CD103^high^PD1^low^ subset) in obese CIA mice (Fig. S5C-D). Collectively, these data provide direct molecular and cellular evidence that obesity exacerbates CIA through hyperactivation of the TGF-β pathway, which is linked to the expansion of pathogenic TRM subsets.

### Asiaticoside alleviated RA by modulating Th17 and TRM cells

**Figure 5:**
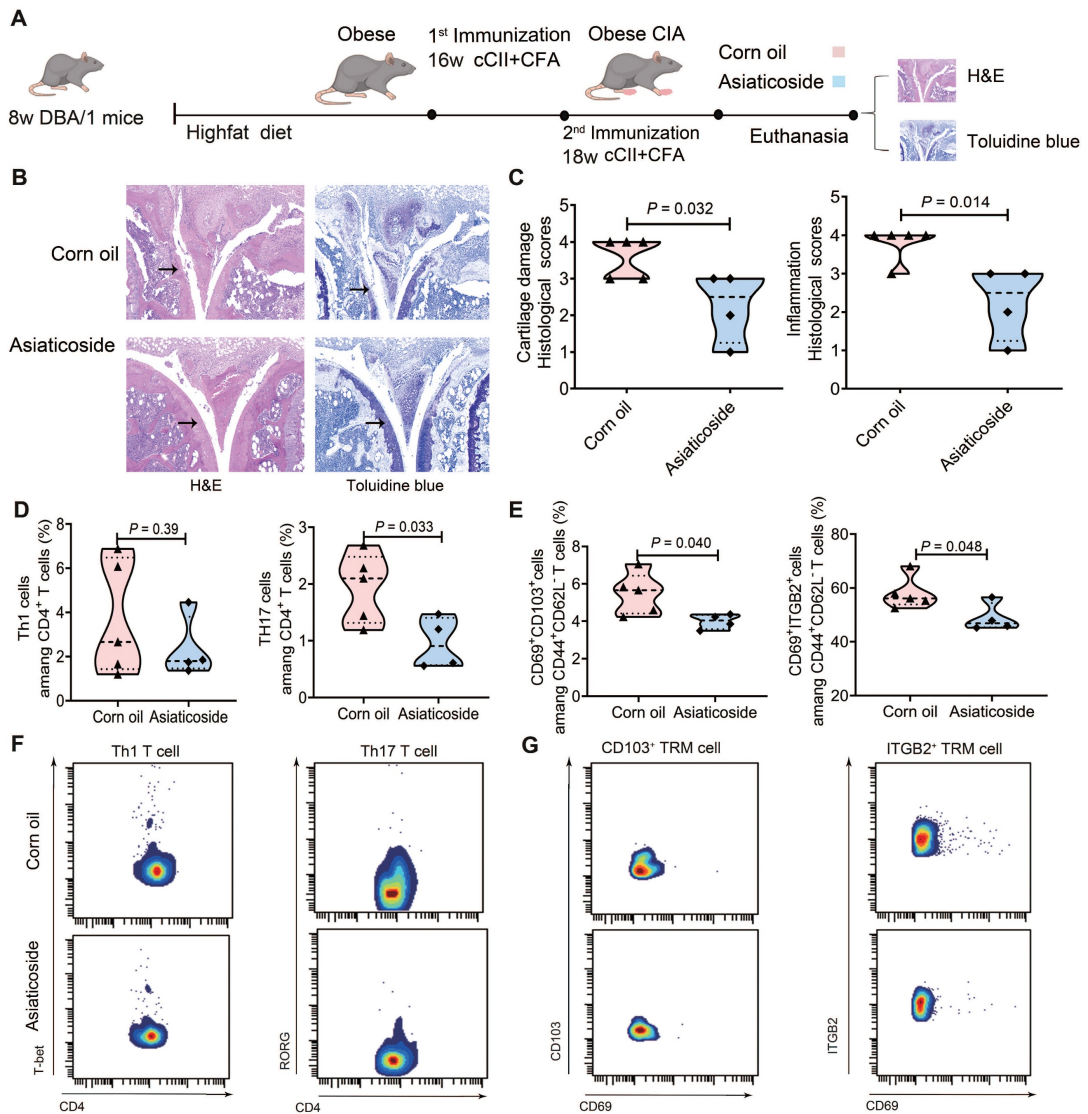
** Asiaticoside alleviated rheumatoid arthritis symptoms in obese CIA mice.** (A) Schematic illustration of high-fat diet, CIA induction, and asiaticoside treatment (20 mg/kg/day, oral gavage) workflows. (B) Representative knee joint sections obtained from obese CIA mice treated with asiaticoside or corn oil (vehicle), stained with H&E (inflammation) and toluidine blue (proteoglycans). Arrows indicate lesions and abnormalities in the joints. Scale bars: 50 μm. (C) Cartilage damage and inflammation histological inflammation scores for the paws of obese CIA mice treated with corn oil (n = 5) and asiaticoside (n = 4), analyzed using the Mann-Whitney U test. (D) Proportion of Th1 (T-bet⁺) and Th17 (RORγt⁺) cells within CD4⁺ T cell in obese CIA mice treated with corn oil (n = 5) or asiaticoside (n = 4), determined using the Mann-Whitney U test. (E) Proportion of CD103⁺ vs ITGB2⁺ TRM subpopulations within CD44⁺CD62L⁻CD69⁺ cells in obese CIA mice treated with corn oil (n = 5) or asiaticoside (n = 4); Mann-Whitney U test was performed for analysis. Each dot represents one mouse. (F-G) Bivariate plots in a paired vertical layout, displaying the asiaticoside-treated obese CIA mice and control obese CIA groups with for each population (Th1 cells, Th17 cells, and TRM phenotypes).

To investigate the role of TGFβRII in Th17 and TRM cell differentiation, we treated mice with asiaticoside, a known inhibitor of TGFβRII/Smad signaling 27, to explore the effects of this pathway in obese CIA mice 17. Asiaticoside (20 mg/kg) or corn oil was administered daily via oral gavage to obese CIA mice for 21 days (Fig. 5A). Body weights at sacrifice remained comparable (46.2 ± 2.7 g vs. 46.4 ± 4.0 g), ruling out off-target metabolic effects. H&E and toluidine blue staining showed that asiaticoside-treated obese CIA mice exhibited reduced synovial inflammation (*P* = 0.032) and cartilage damage (*P* = 0.014) versus control obese CIA mice (Fig. 5B, C).

CyTOF analysis revealed no significant difference in T-bet⁺ Th1 cell proportions but a notable reduction in RORγt⁺ Th17 cells (*P* = 0.033) in the asiaticoside group (Fig. 5D). Concomitantly, the drug decreased CD103⁺ TRM abundance by 27.15% (*P* = 0.040) and ITGB2⁺ TRM abundance by 15.70% (*P* = 0.048) within the CD44⁺CD62L⁻CD69⁺ TRM pool (Fig. 5E). Fig. 5F-G presents bivariate plots in a paired vertical layout displaying the Th1 cell populations, Th17 cell populations, and TRM phenotypes in asiaticoside-treated and control obese CIA mice. This reduction correlated with both the decrease in synovial inflammation and cartilage damage (Fig. 5B-C), directly linking TRM cell suppression to pathological amelioration. These findings confirm that asiaticoside alleviates RA by modulating Th17 cell and TRM subset abundances, whilst CD103⁺ TRM and ITGB2⁺ TRM cells are implicated in pro-inflammatory responses in this obese CIA model.

To functionally characterize the expanded TRM pool, we quantified IFN-γ expression within CD44⁺CD62L⁻ T cell subsets using CyTOF. Normalized IFN-γ protein levels were significantly elevated in obese CIA mice compared with those in lean CIA mice in CD44⁺CD62L⁻CD69⁺ T cells (*P* = 0.017), CD69⁺CD103⁺ TRM cells (*P* = 0.00097), and CD69⁺ITGB2⁺ TRM cells (*P* = 0.0038). Asiaticoside treatment significantly reduced IFN-γ expression in all three subsets in obese CIA mice (*P* < 0.05), directly linking TGFβR hyperactivation to the enhanced pro-inflammatory state of these cells. Together, these data demonstrate that the TGFβR pathway governs not only the numerical expansion but also the functional potency of pathogenic TRM-like and TRM cells in obese CIA mice (Fig. S6).

### Asiaticoside reduced synovial TRM cell abundance

**Figure 6:**
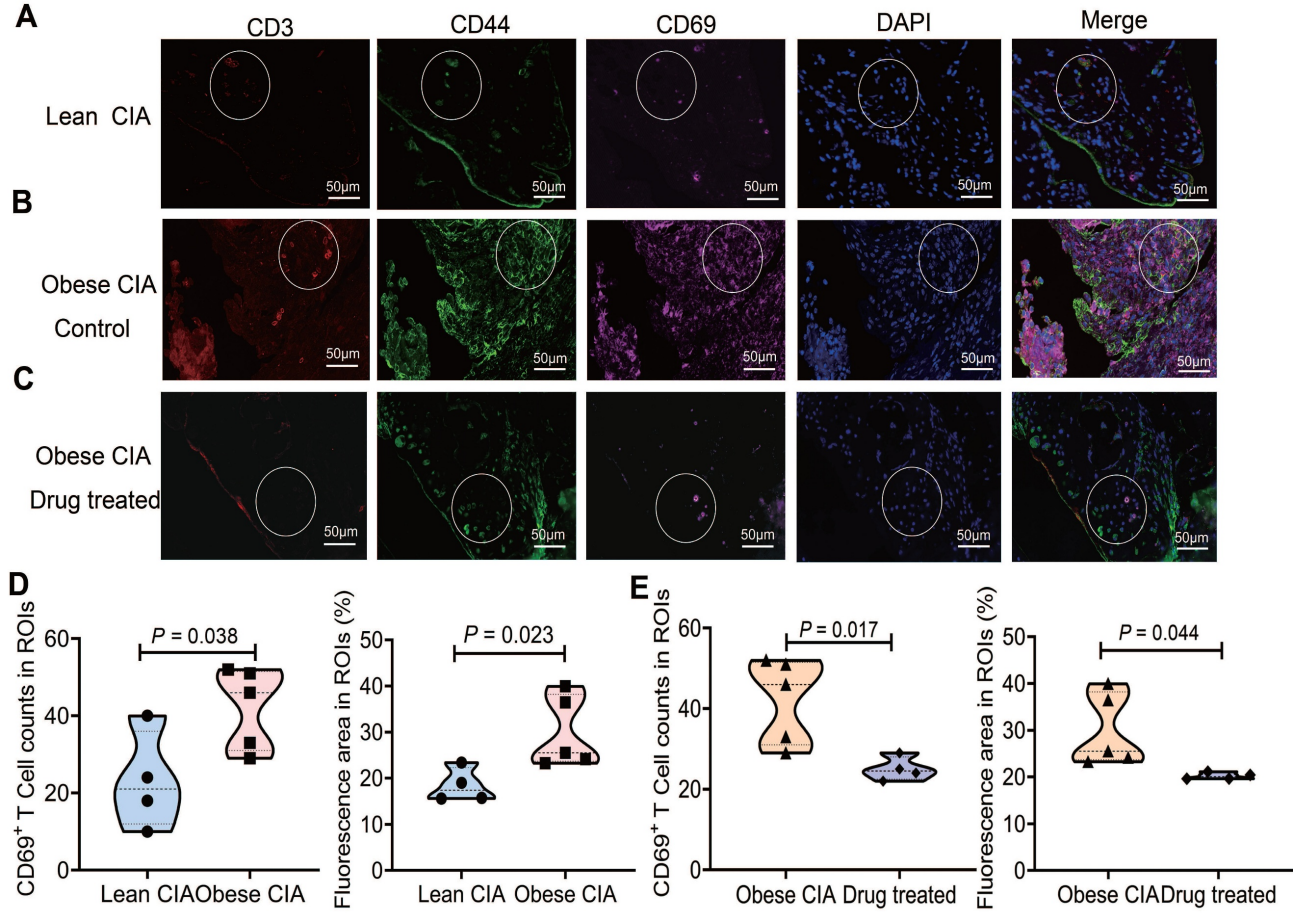
**Asiaticoside treatment reduced synovial TRM accumulation in obese CIA mice.** (A-C) Representative immunofluorescence images of synovium sections co-stained for CD3 (red), CD44 (red), CD69 (cyan), and DAPI (blue) in (A) lean CIA mice, (B) obese CIA mice control (corn oil), and (C) drug-treated obese CIA mice (asiaticoside). White dashed circles indicate regions of interest (ROIs). Scale bars: 50 μm. (D) Density and fluorescence area of synovial TRM cells (CD3⁺CD44⁺CD69⁺) in lean vs. obese CIA mice. (E) Density and fluorescence area within ROIs of vehicle-treated vs. asiaticoside-treated obese CIA mice. Each shape represents one mouse (lean CIA, n = 4; obese CIA mice control, n = 5; obese CIA + asiaticoside, n = 4). Data are presented as the mean ± SD. Statistical comparisons were performed using the Mann-Whitney U test.

To investigate synovial TRM cell distribution in obese and lean CIA mice, we performed multispectral immunofluorescence imaging on synovial samples from lean and obese CIA mice treated with either corn oil or asiaticoside. We assessed T cells for co-expression of CD3, CD44, and CD69, which are core markers of synovial TRM cells (Fig. 6A-C). Compared to lean CIA mice, obese CIA mice showed significantly increased TRM cell numbers and positive staining area within synovial region of interest (ROI). Critically, asiaticoside treatment significantly reduced these measures compared to those in obese controls (Fig. 6D-E). These synovial TRM reductions, combined with decreased circulating and lymph node TRM cells (Fig. 5E), collectively demonstrate that asiaticoside alleviates RA not only by limiting systemic TRM pools but also by suppressing cell accumulation within inflamed joints.

### Preliminary observations suggest enrichment of TRM-like cells in the synovium of patients with a high BMI and RA

To provide pathological context, we evaluated H&E-stained synovial sections from patients with a high or low BMI and RA. Samples from patients with a high BMI (BMI = 32.0) exhibited acute-on-chronic inflammation with fibrinous exudates and structured lymphoid aggregates, indicative of highly active disease. Microscopically, the synovium displayed dense lymphocytic infiltration, tissue edema, and focal connective tissue proliferation. In contrast, the tissues from patients with a low BMI (BMI = 21.3) showed moderate chronic synovitis with a diffuse infiltrate. Its prominent characteristics were connective tissue proliferation, neovascularization, scattered lymphocytic infiltration, and focal hemorrhage (Fig. S7A**-**B). Using tyramide-based immunofluorescence, we detected CD3, CD44, and CD69 in samples from two patients with high BMIs and two with low BMIs (Fig. S7C-D). Consistent with the histopathological findings, the synovium tissues from patients with high BMIs displayed a notable accumulation of CD3⁺CD44⁺CD69⁺ T cells, indicative of TRM. Conversely, triple-positive cells were scarce in the low-BMI samples, with minimal CD69 expression.

Quantitative image analysis across multiple ROIs confirmed a significant increase in CD69-positive areas and TRM-like cell density in high-BMI synovial samples compared with those in low-BMI samples (Fig. S7E). The concurrent observation of more severe histopathology, qualitative enrichment, and a quantitative increase in TRM-like cells in high-BMI samples supports the possibility that obesity may facilitate synovial accumulation, echoing our findings in the murine models. Analysis of a larger patient cohort is underway to statistically confirm this association.

**Figure 7:**
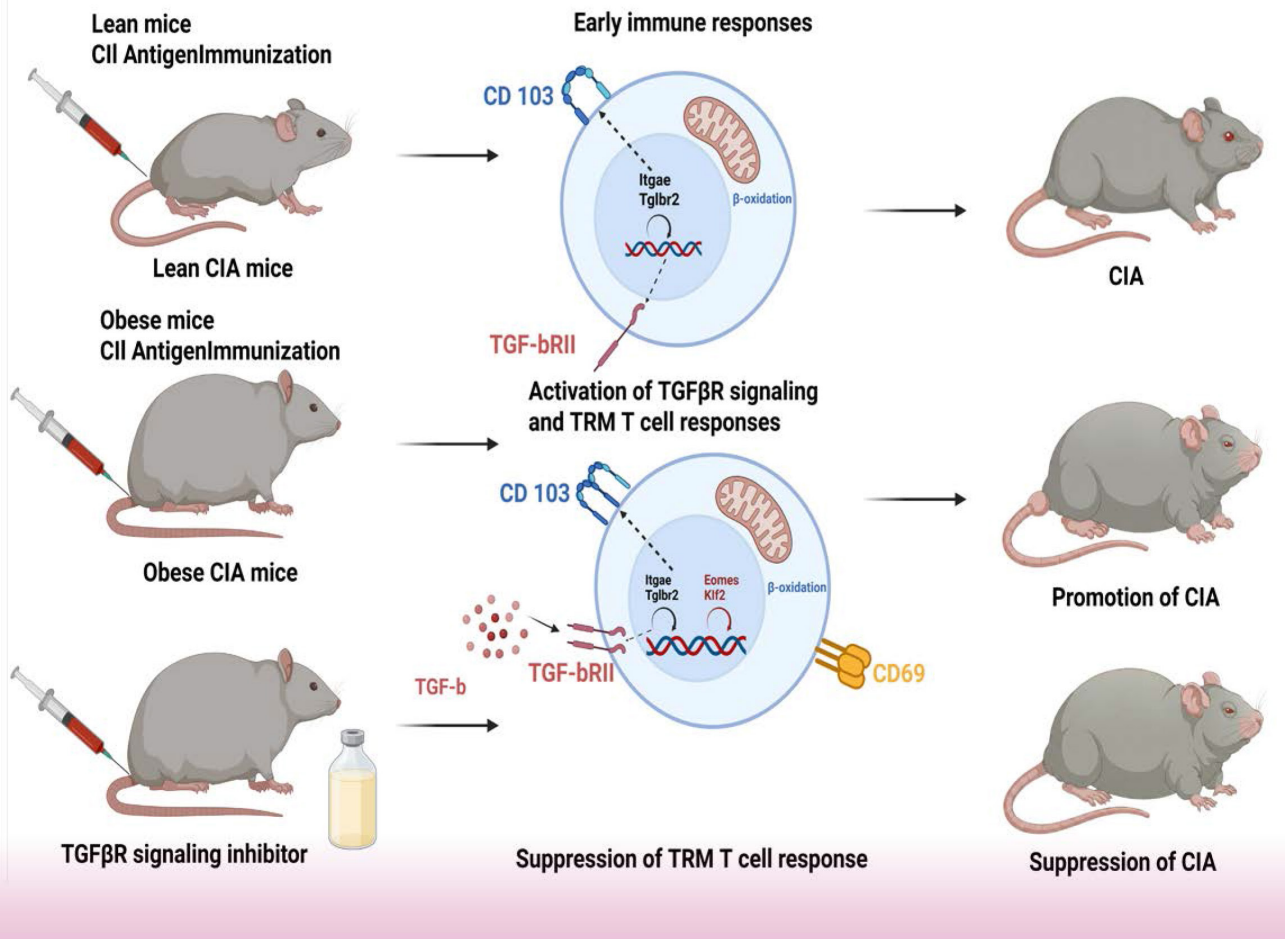
** Schematic model of how the obesity-TGFβ-TRM axis exacerbates collagen-induced arthritis in mice**.

Collectively, our data delineate a pathogenic axis wherein obesity-induced hyperactivation of TGFβ signaling, coupled with suppression of the transcription factors EOMES and KLF2, drives the expansion and synovial accumulation of CD103⁺/ITGB2⁺ TRM cells, ultimately exacerbating arthritis severity. This mechanistic model is summarized in Fig. 7.

## Discussion

The development of RA is largely attributed to an imbalance in immune factors, particularly between anti-inflammatory Tregs and pro-inflammatory Th17 cells 48. Obesity promotes the IL-6-dependent Th17 cell subset bias 49. In CIA mice, obesity exacerbates arthritis symptoms by promoting Th17 T cell differentiation and IL-17 production in the synovium 17. In this study, we used an animal model representing CIA with obesity. Proteomic analysis revealed a significant downregulation of EOMES expression in obese CIA mice compared with that in their lean counterparts. EOMES, a T-box transcription factor, regulates T-cell differentiation by suppressing IL-17/RORγt (inhibiting Th17 differentiation) and promoting IFN-γ (enhancing Th1 effector functions), thereby reinforcing the pro-inflammatory activity of Th1 cells in autoimmune settings 45. A shift from Th1 to Th17 cells as the predominant lymphocytes was confirmed by CyTOF in obese CIA mice, indicating an increase in IL-17 and RORγt expression. Collectively, these findings suggest that suppression of EOMES may contribute to the expansion of Th17 cells and elevated IL-17 levels observed in the joints and spleen cells of obese CIA mice, as previously reported.

TRM cells are widely distributed across various tissues, including the intestine, skin, brain, lungs, female genital tract, salivary glands, thymus, spleen, and lymph nodes, highlighting their role in local immune surveillance. Their functions include local proliferation, production of pro-inflammatory cytokines, induction of dendritic cell maturation, and recruitment of circulating cells. TRM cells have been implicated in triggering autoimmune recurrence and perpetuating diseases, such as fixed drug eruption, allergic airway disease, alopecia areata, inflammatory bowel disease, psoriasis, multiple sclerosis, and RA 50-54. Specifically, the recurrence of autoimmune arthritis in mouse models requires TRM cells within the synovium 55. Synovial TRM cells exhibit long-term residency and preference for fatty acid uptake 56; this metabolic feature aligns with the elevated free fatty acid levels in the joint microenvironment of HFD-induced obese mice, consistent with our hyperlipidemia observation in obese groups (Fig. 1D-F). Through fatty acid uptake and oxidation, TRM cells can sustain their viability and pro-inflammatory effector functions, thereby perpetuating joint inflammation and cartilage damage in obese CIA mice. Transcriptional profiling has revealed that synovial TRM cells are enriched in immune activation and cell recruitment signaling pathways, further supporting their potential role in facilitating RA recurrence through pro-inflammatory effects 55. Furthermore, our data reveal an important evolution in the immunopathological features of arthritis in the obese CIA model, characterized by a decreased proportion of CD4⁺ T cells among CD3⁺ T cells (Fig. 4B), which coincides with an expansion of CD8⁺ T cells and TRM subsets. This suggests that, in the context of obesity, the driving force behind joint injury may shift from a traditional CD4⁺ T cell-dominant response to a more destructive immune attack co-dominated by tissue-resident CD4⁺ and CD8⁺ TRM cells.

After antigen-driven expansion in the lymph nodes, TGF-β is essential for the differentiation and epidermal persistence of skin TRM cells 57. Moreover, TGF-β plays multiple roles in enhancing TRM cell function, including the negative regulation of the transcription factors T-bet, EOMES, and KLF2, which are crucial for T cell trafficking 47, 58. This downregulation is not merely an epiphenomenon but is mechanistically integral to the TRM residency program. KLF2 promotes the expression of trafficking receptors such as S1PR1, which is essential for lymphocyte egress from tissues; therefore, its suppression is a prerequisite for stable tissue residency 58. Similarly, EOMES is associated with effector and circulating memory T cell identities, and its repression helps to solidify the TRM differentiation program, often involving upregulation of residency markers, including CD103 59. Enhanced sensing of TGF-β by CD8^+^ T cells is crucial for tissue residency. Notably, forced TGFβRII expression rescues mitochondrial function deficits in P2RX7-deficient TRM cells 60, further underscoring the importance of this pathway for TRM cell fitness.

Our findings support a "two-hit" model for obesity-exacerbated arthritis. In this model, the first hit (obesity) reprograms the immune baseline and establishes a preconditioned state of the TGF-β signaling axis. This preconditioning manifests as a suppressed receptor state, characterized by downregulated TGFβRII expression in obese control mice compared with that in lean controls, a change that likely represents a compensatory adaptation. Concurrently, KLF2 expression decreases toward inhibition, although the observed decreases were not significant. The second hit (CIA induction) triggers a pathogenic reversal within this preconditioned background, leading to significant upregulation of TGFβRII expression accompanied by further suppression of KLF2 expression. Thus, in the obese CIA model, this synergistic shift from suppression to hyperactivation of the TGFβR pathway, together with deepened inhibition of KLF2 and significant suppression of EOMES, collectively drives the pathological expansion and tissue residency of CD103⁺ TRM cells.

In this study, proteomic profiling of lymphocytes from obese CIA mice demonstrated upregulation of TGFβRII expression alongside alterations in the levels of several TRM markers. This was accompanied by downregulation of EOMES and KLF2 expression. While our data favor a differentiation-driven model for TRM expansion, formally distinguishing this from local proliferation remains an interesting question for future study. Mechanistic insights from other studies connect these factors to immune-metabolic dysregulation. For example, KLF2 deficiency in macrophages, driven by obesity-induced promoter hypermethylation, enhances pro-inflammatory M1 polarization and osteoclast activation, partly via MMP9 upregulation 42, 43. Similarly, EOMES deficiency in T cells can dysregulate the cytotoxic balance (IFN-γ/granzyme B production) and disrupt the Th17/Treg equilibrium by hindering mechanisms such as IL-10/IFN-γ-mediated suppression of Th17 cells 44, 45. In our CyTOF analysis of surface marker expression (CD103, ITGB2, CD69), we identified two distinct populations within the CD44⁺CD62L⁻CD69⁺ TRM pool: CD103⁺ cells exhibited a propensity for enhanced production of pro-inflammatory cytokines (IL-2, TNF-α, IFN-γ), while ITGB2⁺ (CD103⁻) cells showed increased granzyme expression. Importantly, this functional dichotomy was directly validated by our protein expression analyses: we observed a significant increase in IFN-γ expression in the CD69^+^ T cells of obese CIA mice, which was reversed following asiaticoside treatment. Taken together, these in vivo functional data substantiate the pathogenic role of the expanded TRM-like cells. Further ex vivo characterization of sorted synovial TRM cells will be an important step to deepen these functional insights.

Consistent with the proteomic data, the abundances of both TRM subtypes were elevated in obese CIA mice. This increase likely contributes to heightened local cytokine and granzyme levels within the joints, potentially exacerbating chondrocyte dysfunction and leading to more severe cartilage damage 61. Supporting a potential pathogenic role for TRM cells, treatment with asiaticoside, which reduced the frequency of these cells in obese CIA mice, was associated with alleviation of arthritis symptoms. These results further imply that asiaticoside may exert its anti-arthritic effects partly by targeting the obesity-TGFβ-TRM axis identified in this study.

Additionally, the observed obesity-induced enhancement of TGFβRII signaling and suppression of EOMES and KLF2 might influence other immune cells beyond TRM and Th17, such as macrophages and B cells. Production of IL-2 and TNF-α plays an important role in modulating various T cell responses. Notably, TGF-β is also a key factor in the differentiation of IL-17-secreting Th17 cell subsets 57. Furthermore, asiaticoside-mediated reduction in TRM cell frequency correlated with attenuated arthritis severity. Importantly, the effect of asiaticoside may not be limited to TRM cells. Preclinical studies suggest that asiaticoside can modulate the function of other immune cells involved in RA. For instance, asiaticoside can inhibit the MAPK and NF-κB signaling pathways and the production of pro-inflammatory cytokines (e.g., IL-6, TNF-α) in macrophages 62. Furthermore, asiaticoside can suppress TGF-β/Smad signaling by inducing Smad7 and inhibiting TGFβRI/II in keloid fibroblasts 63. A comprehensive review highlighted that after oral administration to rats, asiaticoside is absorbed and distributed to various tissues, and its efficient lipophilicity suggests potential for distribution to other sites of inflammation 64. While studies of asiaticoside in human RA are still needed, highly relevant translational data exist for its active metabolite, asiatic acid. Recent studies have demonstrated that asiatic acid inhibits the proliferation of and induces ferroptosis in fibroblast-like synoviocytes in human RA through the Nrf2/HO-1 pathway, and alleviates inflammation and joint damage in a CIA rat model 65, 66. Notably, beyond the Smad-dependent pathway, TGF-β can also act through several Smad-independent pathways, including MAPK pathways (such as p38, JNK, and ERK), PI3K-Akt, and Rho-like GTPase 67, 68. In addition, in the context of the pro-inflammatory milieu of RA, non-canonical pathways can contribute to synovial fibroblast activation and osteoclast differentiation, thereby perpetuating joint destruction 69, 70. However, the precise pathways through which asiaticoside targets TRM cells in this context require further validation. While our phenotypic and correlative data strongly support the involvement of the TGF-β/TRM axis, direct biochemical validation of asiaticoside's inhibition of Smad2/3 phosphorylation in this specific model represents an important direction for future investigation.

Our findings reveal a novel obesity-TGFβ-TRM axis through which CIA is exacerbated. This mechanism may have broader implications for autoimmune diseases beyond RA. Pathogenic TRM cells are recognized as key drivers of chronicity and recurrence in psoriasis and multiple sclerosis 71, 72. The conserved role of TRM cells across these conditions suggests that the axis we identified may represent a common pathway for disease exacerbation. Consequently, TGFβR and TRM cells emerge as promising therapeutic targets for a spectrum of TRM-mediated autoimmune conditions, particularly those associated with obesity.

Despite the promising findings, our study has some limitations. First, we acknowledge that CIA induction at 16 weeks of age represents a limitation, as this age approaches the upper limit of the optimal window for the DBA/1 model 21. This age of induction was selected to accommodate the prior dietary intervention period, and we ensured that the critical comparison demonstrating obesity-driven exacerbation was made between age-matched groups. However, the potential confounding effect of advanced age should be considered in the interpretation of our results. Second, in vivo TRM depletion experiments to formally establish a causal role for synovial TRM cells in obese CIA were not conducted. To directly establish the causal role of TRM cells, subsequent investigations could employ inducible CD103-dependent depletion models, a strategy recently proven effective in elucidating TRM function in other tissues. Third, due to the 12-week period required for HFD-induced obesity modeling, we were unable to supplement all groups simultaneously within the revision timeframe. Statistical validation confirmed comparability between the two lean CIA cohorts (P > 0.05 for all key parameters), and data sources are transparently reported in the figure legends. However, we acknowledge that minor batch effects cannot be completely excluded, and future studies with concurrently enrolled cohorts would further validate our findings. Fourth, the absence of an “asiaticoside-only” control group precludes definitive conclusions regarding potential off-target effects of asiaticoside on the obesity-primed immune milieu, independent of arthritis. Future studies incorporating this control will be valuable to fully determine the drug's mechanism of action.

Furthermore, the direct regulation of the TGFβR-TRM axis by obesity-related metabolic signals (e.g., free fatty acids) remains unexplored. Notably, recent research has demonstrated significant fatty acid metabolic reprogramming in T cells from patients with RA; T cell pro-inflammatory functions are dependent on fatty acid uptake and oxidation 73. Thus, elucidating how metabolic dysregulation in the obese milieu interfaces with TGFβR signaling to drive TRM pathology represents a critical future direction. While the mouse model used in this study is invaluable for mechanistic discoveries, it may not recapitulate all aspects of human RA pathogenesis. Notably, the DBA/1 line, although optimal for CIA induction, exhibits a metabolic response to HFDs that differs from other models, such as C57BL/6 mice, and therefore may not comprehensively mirror human obesity. Encouragingly, studies have confirmed that a CD8⁺-predominant TRM population, phenotypically like the subsets identified here, is present in the human RA synovium and is enriched in late-stage, non-inflamed tissues, suggesting its role as a key mediator of disease chronicity 55.

In this study, our correlative analysis of synovial tissues from patients with RA was preliminary, and the sample size was limited. This is primarily due to the relative scarcity of obese patients meeting the study criteria within the Chinese RA population, coupled with a constrained timeframe for sample collection. This constitutes a clear limitation, precluding robust statistical conclusions or adjustment for clinical confounders. Therefore, formal validation of the presence, metabolic features, and pathogenic role of TRM subsets in the synovial tissues of obese patients with RA is crucial to provide definitive translational validation for our findings.

In conclusion, we identified a novel obesity-TGFβ-TRM pathogenic axis, wherein suppressed EOMES/KLF2 expression and hyperactive TGFβ signaling collectively drive the pathological expansion of Th17 cells and tissue-resident TRM populations, thereby exacerbating collagen-induced arthritis. The obese CIA mouse model represents a valuable platform for dissecting the immunometabolic crosstalk between obesity and inflammatory arthritis, enabling the identification of tractable therapeutic targets including TGFβRII and EOMES/KLF2-associated signaling pathways. For translational applications, our findings support the repurposing of TGFβRII-targeted monoclonal antibodies (e.g., fresolimumab), currently under clinical investigation for fibrosis, to disrupt pathogenic TRM residency in RA. More innovatively, this work highlights the promise of precision therapies targeting fatty acid metabolism specifically for patients with obesity-associated RA. To advance clinical translation, future studies should prioritize single-cell multi-omics to define a distinct “high-TRM” pathogenic subtype in obese patients with RA. Characterization of unique surface markers and metabolic receptors on these pathogenic TRM cells will enable patient stratification and the development of targeted biologic or metabolic therapies for this high-risk population. Ultimately, the design of novel pharmacotherapies that concomitantly target inflammatory pathways and underlying metabolic dysregulation represents a promising strategy to address the unmet therapeutic challenges of obesity-related rheumatoid arthritis. This newly identified immunometabolic axis thus provides a mechanistic and translational framework for advancing precision medicine against obesity-linked autoimmune arthritis.

## Supplementary Material

Supplementary methods and figures.

Supplementary tables.

## Figures and Tables

**Table 1:** The 26 DEPs in obese and lean CIA mice lymphocytes

Protein name	Obese CIA	Lean CIA	Fold change	*P*-value
IGHG	308405	38931	7.92	0.010
MCPT4	9703180	2158053	4.50	0.005
CMA1	4528940	1061710	4.27	0.015
CPA3	3884020	1084738	3.58	0.008
UCP1	351953	110541	3.18	0.025
MRPL55	141864	49234	2.88	0.034
FUNDC2	927388	360373	2.57	0.013
CIRBP	1145724	569373	2.01	0.013
ITGAE(CD103)	180190	96225	1.87	0.050
USE1	109283	62708	1.74	0.019
MTCO1	849008	495693	1.71	0.033
APOE	191684	114089	1.68	0.013
FCGR2(CD32)	633086	408123	1.55	0.001
UBASH3B	87808	57675	1.52	0.015
SELENOF	635728	423415	1.50	0.038
CCNL1	59621	138883	0.43	0.001
KLF2	47808	108145	0.44	0.042
DOP1B	70397	157420	0.45	0.030
HSD11B1	319204	700218	0.46	0.001
EOMES	39444	81144	0.49	0.011
BNIP1	438116	819030	0.54	0.044
MOCS2	315120	548093	0.58	0.008
UQCC2	92288	158377	0.58	0.031
S100A13	665660	1098753	0.61	0.017
WDR6	100687	161828	0.62	0.037
MOV10	491830	749440	0.66	0.017

**Table 2:** Annotation of immune cell metaclusters identified using FlowSOM analysis in RA mouse lymph nodes

Metacluster	Cell Type	Key Phenotype*
M1	Resting Effector Memory T Cells	CD3⁺, CD44⁺, CD62L⁻, ITGB2⁺, CD69⁻
M2	NK/NKT Cells	CD16⁺⁺, CD3⁺, CD8A⁺, CD69⁻
M3	Early Activated CD4⁺ T Cells	CD69⁺⁺⁺, CD4⁺, CD3⁺, CD44⁻/^low^
M4	Hyperactivated Double-Positive T Cells	CD69⁺⁺⁺⁺, CD4⁺, CD8A⁺, CD16⁺⁺
M5	Tregs Cells	FOXP3⁺⁺, CD3⁺⁺, CD25⁻/^low^
M6	CD25⁺ Innate-like/Activated Lymphocytes	CD25⁺⁺⁺, CD69⁺, CD3⁻/^low^
M7	Th1 Cells	T-bet⁺⁺⁺⁺, CD62L⁺⁺, CD3⁺
M8	Activated Effector Memory T Cells	CD3E⁺⁺⁺⁺, CD44⁺⁺, CD161⁺, ITGB2⁺
M9	Th17 Cells	RORγt⁺⁺⁺, CD161⁺⁺⁺, CCR7⁺⁺, ITGB2⁺⁺⁺
M10	CD103⁺ TRM Cells	CD103⁺, CD69⁺⁺, CD25⁺⁺⁺, CD44⁺
M11	ITGB2⁺ TRM like Cells	CD44⁺⁺⁺⁺, ITGB2⁺⁺, CD3⁺⁺, CD103⁻
M12	CCR7⁺ RORγt⁺ T Cells (Th17-like Tcm)	RORγt⁺⁺, CCR7⁺, CD161⁺, CD44⁺

*-Negative/Very Low, + : Low, ++: Moderate, +++: High, ++++: Very High

## Data Availability

The datasets used and/or analyzed during the current study are available from the corresponding author upon reasonable request.

## Abbreviations

RA: rheumatoid arthritis
HFD: high-fat diet
CIA: collagen-induced arthritis
CyTOF: cytometry by time-of-flight
TRM: tissue-resident memory
CII: type II collagen
MS: mass spectrometry
ND: normal diet
CFA: Complete Freund's adjuvant
MRI: magnetic resonance imaging
FDR: false discovery rate
DEP: differentially expressed proteins
KEGG: Kyoto Encyclopedia of Genes and Genomes
GO: gene ontology
MF: molecular functions
CC: cellular components
BP: biological processes
RAS: renin-angiotensin system
MCs: mast cells
MFI: mean fluorescence intensity
viSNE: variational inference for stochastic neighbor embedding
LAP: latency-associated peptide
ROI: region of interest
DDA: data-dependent acquisition

## References

[1] McInnes IB, Schett G (2011). The Pathogenesis of Rheumatoid Arthritis. The New England Journal of Medicine.

[2] Dadoun S, Zeboulon-Ktorza N, Combescure C, Elhai M, Rozenberg S, Gossec L (2013). Mortality in rheumatoid arthritis over the last fifty years: Systematic review and meta-analysis. Joint Bone Spine.

[3] Meune C, Touzé E, Trinquart L, Allanore Y (2009). Trends in cardiovascular mortality in patients with rheumatoid arthritis over 50 years: a systematic review and meta-analysis of cohort studies. Rheumatology.

[4] Chung HY, Cesari M, Anton S, Marzetti E, Giovannini S, Seo AY (2009). Molecular inflammation: underpinnings of aging and age-related diseases. Ageing Res Rev.

[5] Helmick CG, Felson DT, Lawrence RC, Gabriel S, Hirsch R, Kwoh CK (2008). Estimates of the prevalence of arthritis and other rheumatic conditions in the United States: Part I. Arthritis & Rheumatism.

[6] Symmons DPM, Bankhead CR, Harrison BJ, Brennan P, Silman AJ, Barrett EM (1997). Blood transfusion, smoking, and obesity as risk factors for the development of rheumatoid arthritis. Results from a primary care-based incident case-control study in Norfolk, England. Arthritis & Rheumatism.

[7] Rodrigues AM, Reis JE, Santos C, Pereira MP, Loureiro C, Martins F (2014). A1.1 Obesity is a risk factor for worse treatment response in rheumatoid arthritis patients- results from reuma.pt. Annals of the Rheumatic Diseases.

[8] Liu Y, Hazlewood GS, Kaplan GG, Eksteen B, Barnabe C (2017). Impact of Obesity on Remission and Disease Activity in Rheumatoid Arthritis: A Systematic Review and Meta-Analysis. Arthritis Care & Research.

[9] Friedman JM, Halaas JL (1998). Leptin and the regulation of body weight in mammals. Nature.

[10] Scherer PE, Williams S, Fogliano M, Baldini G, Lodish HF (1995). A Novel Serum Protein Similar to C1q, Produced Exclusively in Adipocytes (*). Journal of Biological Chemistry.

[11] Weisberg SP (2003). Obesity is associated with macrophage accumulation in adipose tissue. J Clin Invest.

[12] Lago F, Dieguez C, Gómez-Reino J, Gualillo O (2007). The emerging role of adipokines as mediators of inflammation and immune responses. Cytokine & Growth Factor Reviews.

[13] Gremese E, Tolusso B, Gigante MR, Ferraccioli G (2014). Obesity as a Risk and Severity Factor in Rheumatic Diseases (Autoimmune Chronic Inflammatory Diseases). Frontiers in immunology.

[14] Farrag Y, Farrag M, Varela-García M, Torrijos-Pulpón C, Capuozzo M, Ottaiano A (2024). Adipokines as potential pharmacological targets for immune inflammatory rheumatic diseases: Focus on rheumatoid arthritis, osteoarthritis, and intervertebral disc degeneration. Pharmacological Research.

[15] McInnes IB, Buckley CD, Isaacs JD (2016). Cytokines in rheumatoid arthritis — shaping the immunological landscape. Nature Reviews Rheumatology.

[16] Shoda H, Nagafuchi Y, Tsuchida Y, Sakurai K, Sumitomo S, Fujio K (2017). Increased serum concentrations of IL-1 beta, IL-21 and Th17 cells in overweight patients with rheumatoid arthritis. Arthritis Research & Therapy.

[17] Jhun J-Y, Yoon B-Y, Park M-K, Oh H-J, Byun J-K, Lee S-Y (2012). Obesity aggravates the joint inflammation in a collagen-induced arthritis model through deviation to Th17 differentiation. Experimental & Molecular Medicine.

[18] Asquith DL, Miller AM, McInnes IB, Liew FY (2009). Animal models of rheumatoid arthritis. European Journal of Immunology.

[19] Caplazi P, Baca M, Barck K, Carano RAD, DeVoss J, Lee WP (2015). Mouse Models of Rheumatoid Arthritis. Veterinary Pathology.

[20] Percie du Sert N, Ahluwalia A, Alam S, Avey MT, Baker M, Browne WJ (2020). Reporting animal research: Explanation and elaboration for the ARRIVE guidelines 2.0. PLOS Biology.

[21] Brand DD, Latham KA, Rosloniec EF (2007). Collagen-induced arthritis. Nature Protocols.

[22] Inglis JJ, Šimelyte E, McCann FE, Criado G, Williams RO (2008). Protocol for the induction of arthritis in C57BL/6 mice. Nature Protocols.

[23] Zhang Z, Li X, Li D, Luo M, Li Y, Song L (2017). Asiaticoside ameliorates β-amyloid-induced learning and memory deficits in rats by inhibiting mitochondrial apoptosis and reducing inflammatory factors. Exp Ther Med.

[24] Xinghai L, Tong L, Kunli Y, Hong L (2023). Antifatigue Effect of Asiaticoside in Mice by Attenuating Oxidative Stress. Discovery Medicine.

[25] Wiśniewski JR, Zougman A, Nagaraj N, Mann M (2009). Universal sample preparation method for proteome analysis. Nature Methods.

[26] Cox J, Mann M (2008). MaxQuant enables high peptide identification rates, individualized p.p.b.-range mass accuracies and proteome-wide protein quantification. Nature Biotechnology.

[27] Cox J, Hein MY, Luber CA, Paron I, Nagaraj N, Mann M (2014). Accurate Proteome-wide Label-free Quantification by Delayed Normalization and Maximal Peptide Ratio Extraction, Termed MaxLFQ*. Molecular & Cellular Proteomics.

[28] Cox J, Hein MY, Luber CA, Paron I, Nagaraj N, Mann M (2014). Accurate proteome-wide label-free quantification by delayed normalization and maximal peptide ratio extraction, termed MaxLFQ. Mol Cell Proteomics.

[29] Mitchell AL, Attwood TK, Babbitt PC, Blum M, Bork P, Bridge A (2019). InterPro in 2019: improving coverage, classification and access to protein sequence annotations. Nucleic Acids Research.

[30] Bendall Sean C, Simonds Erin F, Qiu P, Amir El-ad D, Krutzik Peter O, Finck R (2011). Single-Cell Mass Cytometry of Differential Immune and Drug Responses Across a Human Hematopoietic Continuum. Science.

[31] Ornatsky OI, Kinach R, Bandura DR, Lou X, Tanner SD, Baranov VI (2008). Development of analytical methods for multiplex bio-assay with inductively coupled plasma mass spectrometry. Journal of Analytical Atomic Spectrometry.

[32] Finck R, Simonds EF, Jager A, Krishnaswamy S, Sachs K, Fantl W (2013). Normalization of mass cytometry data with bead standards. Cytometry Part A.

[33] Levine Jacob H, Simonds Erin F, Bendall Sean C, Davis Kara L, Amir E-ad D, Tadmor Michelle D (2015). Data-Driven Phenotypic Dissection of AML Reveals Progenitor-like Cells that Correlate with Prognosis. Cell.

[34] Gaudillière B, Fragiadakis GK, Bruggner RV, Nicolau M, Finck R, Tingle M (2014). Clinical recovery from surgery correlates with single-cell immune signatures. Science Translational Medicine.

[35] Chen H, Lau MC, Wong MT, Newell EW, Poidinger M, Chen J (2016). Cytofkit: A Bioconductor Package for an Integrated Mass Cytometry Data Analysis Pipeline. PLOS Computational Biology.

[36] Faul F, Erdfelder E, Buchner A, Lang A-G (2009). Statistical power analyses using G*Power 3.1: Tests for correlation and regression analyses. Behavior Research Methods.

[37] Benjamini Y, Hochberg Y (1995). Controlling the False Discovery Rate: A Practical and Powerful Approach to Multiple Testing. Journal of the Royal Statistical Society: Series B (Methodological).

[38] The Gene Ontology Consortium (2019). The Gene Ontology Resource: 20 years and still GOing strong. Nucleic acids research.

[39] Kanehisa M, Furumichi M, Tanabe M, Sato Y, Morishima K (2017). KEGG: new perspectives on genomes, pathways, diseases and drugs. Nucleic Acids Research.

[40] Price A, Lockhart JC, Ferrell WR, Gsell W, McLean S, Sturrock RD (2007). Angiotensin II type 1 receptor as a novel therapeutic target in rheumatoid arthritis: In vivo analyses in rodent models of arthritis and ex vivo analyses in human inflammatory synovitis. Arthritis & Rheumatism.

[41] Suurmond J, Habets KLL, Dorjée AL, Huizinga TW, Toes REM (2016). Expansion of Th17 Cells by Human Mast Cells Is Driven by Inflammasome-Independent IL-1β. The Journal of Immunology.

[42] Das M, Deb M, Laha D, Joseph M, Kanji S, Aggarwal R (2019). Myeloid Krüppel-Like Factor 2 Critically Regulates K/BxN Serum-Induced Arthritis. Cells.

[43] Sweet DR, Vasudevan NT, Fan L, Booth CE, Keerthy KS, Liao X (2020). Myeloid Krüppel-like factor 2 is a critical regulator of metabolic inflammation. Nat Commun.

[44] Geginat J, Vasco C, Gruarin P, Bonnal R, Rossetti G, Silvestri Y (2023). Eomesodermin-expressing type 1 regulatory (EOMES+Tr1)-like T cells: Basic biology and role in immune-mediated diseases. European Journal of Immunology.

[45] Mazzoni A, Maggi L, Siracusa F, Ramazzotti M, Rossi MC, Santarlasci V (2019). Eomes controls the development of Th17-derived (non-classic) Th1 cells during chronic inflammation. European Journal of Immunology.

[46] Watanabe R, Kadoba K, Tamamoto A, Murata K, Murakami K, Onizawa H (2023). CD8+ Regulatory T Cell Deficiency in Elderly-Onset Rheumatoid Arthritis. Journal of Clinical Medicine.

[47] Mackay Laura K, Wynne-Jones E, Freestone D, Pellicci Daniel G, Mielke Lisa A, Newman Dane M (2015). T-box Transcription Factors Combine with the Cytokines TGF-β and IL-15 to Control Tissue-Resident Memory T Cell Fate. Immunity.

[48] Al-Zifzaf DS, El Bakry SA, Mamdouh R, Shawarby LA, Ghaffar AYA, Amer HA (2015). FoxP3+T regulatory cells in Rheumatoid arthritis and the imbalance of the Treg/TH17 cytokine axis. The Egyptian Rheumatologist.

[49] Winer S, Paltser G, Chan Y, Tsui H, Engleman E, Winer D (2009). Obesity predisposes to Th17 bias. European Journal of Immunology.

[50] Cheuk S, Wikén M, Blomqvist L, Nylén S, Talme T, Ståhle M (2014). Epidermal Th22 and Tc17 cells form a localized disease memory in clinically healed psoriasis. J Immunol.

[51] Cheuk S, Schlums H, Gallais Sérézal I, Martini E, Chiang SC, Marquardt N (2017). CD49a Expression Defines Tissue-Resident CD8+ T Cells Poised for Cytotoxic Function in Human Skin. Immunity.

[52] Kleinschek M, Boniface K, Sadekova S, Faubion W, de Waal Malefyt R, Pierce R (2009). Circulating and Gut-resident Human Th17 Cells Express CD161 and Promote Intestinal Inflammation. Clinical Immunology.

[53] Sasaki K, Bean A, Shah S, Schutten E, Huseby PG, Peters B (2014). Relapsing-remitting central nervous system autoimmunity mediated by GFAP-specific CD8 T cells. J Immunol.

[54] Sherlock JP, Joyce-Shaikh B, Turner SP, Chao C-C, Sathe M, Grein J (2012). IL-23 induces spondyloarthropathy by acting on ROR-γt+ CD3+CD4-CD8- entheseal resident T cells. Nature Medicine.

[55] Chang MH, Levescot A, Nelson-Maney N, Blaustein RB, Winden KD, Morris A (2021). Arthritis flares mediated by tissue-resident memory T cells in the joint. Cell Reports.

[56] Pan Y, Tian T, Park CO, Lofftus SY, Mei S, Liu X (2017). Survival of tissue-resident memory T cells requires exogenous lipid uptake and metabolism. Nature.

[57] Hirai T, Yang Y, Zenke Y, Li H, Chaudhri VK, De La Cruz Diaz JS (2021). Competition for Active TGFβ Cytokine Allows for Selective Retention of Antigen-Specific Tissue- Resident Memory T Cells in the Epidermal Niche. Immunity.

[58] Skon CN, Lee J-Y, Anderson KG, Masopust D, Hogquist KA, Jameson SC (2013). Transcriptional downregulation of S1pr1 is required for the establishment of resident memory CD8+ T cells. Nature Immunology.

[59] Zhang N, Bevan Michael J (2013). Transforming Growth Factor-β Signaling Controls the Formation and Maintenance of Gut-Resident Memory T Cells by Regulating Migration and Retention. Immunity.

[60] Borges da Silva H, Peng C, Wang H, Wanhainen KM, Ma C, Lopez S (2020). Sensing of ATP via the Purinergic Receptor P2RX7 Promotes CD8+ Trm Cell Generation by Enhancing Their Sensitivity to the Cytokine TGF-β. Immunity.

[61] Schuerwegh AJ, Dombrecht EJ, Stevens WJ, Van Offel JF, Bridts CH, De Clerck LS (2003). Influence of pro-inflammatory (IL-1α, IL-6, TNF-α, IFN-γ) and anti-inflammatory (IL-4) cytokines on chondrocyte function. Osteoarthritis and Cartilage.

[62] Shin HY, Kim YS, Ha EJ, Koo JP, Jeong WB, Joung MY (2024). Anti-inflammatory action and associated intracellular signaling of Centella asiatica extract on lipopolysaccharide-stimulated RAW 264.7 macrophage. Food Bioscience.

[63] Tang B, Zhu B, Liang Y, Bi L, Hu Z, Chen B (2011). Asiaticoside suppresses collagen expression and TGF-β/Smad signaling through inducing Smad7 and inhibiting TGF-βRI and TGF-βRII in keloid fibroblasts. Archives of Dermatological Research.

[64] He Z, Hu Y, Niu Z, Zhong K, Liu T, Yang M (2023). A review of pharmacokinetic and pharmacological properties of asiaticoside, a major active constituent of Centella asiatica (L.) Urb. Journal of Ethnopharmacology.

[65] Sun M, Wang Q, Huang J, Sun Q, Yu Q, Liu X (2024). Asiatic acid induces ferroptosis of RA-FLS via the Nrf2/HMOX1 pathway to relieve inflammation in rheumatoid arthritis. International Immunopharmacology.

[66] Zhang L, Liu Z-n, Han X-y, Liu X, Li Y (2024). Asiatic acid inhibits rheumatoid arthritis fibroblast-like synoviocyte growth through the Nrf2/HO-1/NF-κB signaling pathway. Chemical Biology & Drug Design.

[67] Derynck R, Zhang YE (2003). Smad-dependent and Smad-independent pathways in TGF-β family signalling. Nature.

[68] Zhang YE (2009). Non-Smad pathways in TGF-β signaling. Cell Research.

[69] Bottini N, Firestein GS (2013). Duality of fibroblast-like synoviocytes in RA: passive responders and imprinted aggressors. Nature Reviews Rheumatology.

[70] Duan M, Wang Q, Liu Y, Xie J (2021). The role of TGF-β2 in cartilage development and diseases. Bone & Joint Research.

[71] Deng G, Zhang Y, Song J, Zhang Y, Zheng Q, Luo Y (2024). The role and therapeutic strategies for tissue-resident memory T cells, central memory T cells, and effector memory T cells in psoriasis. Immunology.

[72] Pignata A, Frieser D, Gonzalez-Fierro C, Hsiao C-C, Engelenburg HJ, Alis M (2025). Tissue-resident memory CD4+ T cells infiltrate the CNS in progressive multiple sclerosis and contribute to chronic autoimmunity in mice. Science Translational Medicine.

[73] Kraus FV, Keck S, Klika KD, Graf J, Carvalho RA, Lorenz H-M (2023). Reduction of Proinflammatory Effector Functions Through Remodeling of Fatty Acid Metabolism in CD8+ T Cells from Rheumatoid Arthritis Patients. Arthritis & Rheumatology.

