# Supplementary material for: TGFβR Hyperactivation Drives CD103⁺/ITGB2⁺ TRM Cell Accumulation in High-Fat Diet-Exacerbated CIA
Source: Int J Biol Sci. 2026 Mar 30;22(8):4095–115. doi: 10.7150/ijbs.124386 (PMC13137863; doi:10.7150/ijbs.124386)
Supplement: Supplementary file 1 — Supplementary methods and figures. [file ijbsv22p4095s1.pdf]

Supplementary Materials

Supplemental Methods

1. Clinical evaluation of CIA

The severity of CIA was evaluated by three independent observers. Observations of the onset and severity were conducted twice weekly for 20 weeks following the second immunization. The severity of arthritis was assessed in individual mouse on a scale of 0–4 according to the following criteria.

Table S1. Scoring system of CIA

Score	Clinical observations
0	No edema or swelling
1	Mild edema and erythema limited to the foot or ankle
2	Mild edema and erythema from the ankle to tarsal
3	Moderate edema and erythema from the ankle to tarsal
4	Edema and erythema from the ankle to the whole leg

Arthritis scores for each mouse were calculated as the sum of the scores from four limbs.

Table S2. Final group assignment after randomization and the corresponding arthritis induction results following the second immunization.

Individual mouse*	Treatment	Day of onset	Score at onset	End score	End weight
Lean 1	CFA	N/A	0	0	28
Lean 2	CFA	N/A	0	0	28
Lean 3	CFA	N/A	0	0	26
Lean 4	CFA	N/A	0	0	31
Lean 5	CFA	N/A	0	0	30
Lean 6	CII+CFA	24	2	11	27
Lean 7	CII+CFA	23	3	12	27
Lean 8	CII+CFA	25	2	12	27
Lean 9	CII+CFA	27	3	14	25
Obese 1	CFA	N/A	0	0	49
Obese 2	CFA	N/A	0	0	47
Obese 3	CFA	N/A	0	0	48
Obese 4	CFA	N/A	0	0	38
Obese 5	CFA	N/A	0	0	46
Obese 6	CII+CFA	25	3	14	44
Obese 7	CII+CFA	22	2	15	45
Obese 8	CII+CFA	23	3	14	43
Obese 9	CII+CFA	24	4	14	40
Obese 10	CII+CFA	25	3	16	52

*: Mice were randomly assigned to groups as detailed in the Methods section. The ear tag numbers listed represent the final composition of each group after the randomization procedure was completed and are provided for data traceability. Group assignment was not based on the order of ear tag numbers. As confirmed in Supplementary Fig. S1A, there were no significant differences in body weight between the Obese CIA and Obese Control groups, or between the Lean CIA and Lean Control groups, demonstrating the success of the randomization.

2. Biochemical parameters

After the mice were sacrificed, the serum was retained for biochemical detection. Total serum cholesterol, glucose, and triglyceride levels were measured using an automatic biochemical analyzer (Redu Life Technology, Shenzhen, Guangdong, China).

3. Histopathological analysis of arthritis

Histopathologic changes were evaluated using hematoxylin and eosin (H&E)–stained and toluidine blue–stained sections by two independent observers in a blinded manner. Inflammation and bone erosion were scored on the sections stained with H&E according to the following criteria:

Table S3. Scoring system of inflammation

Score	Clinical observations
0	No inflammation
1	Slight thickening of the lining layer or some infiltrating cells in the underlying layer
2	Slight thickening of the lining layer and some infiltrating cells in the underlying layer
3	Thickening of the lining layer, an influx of underlying cells, and presence of cells in the synovial space
4	Extensive infiltration of the synovium with numerous inflammatory cells

Cartilage damage was assessed on the sections stained with toluidine blue according to the following criteria:

Table S4. Scoring system of cartilage damage

Score	Clinical observations
0	No damage
1	Minimal erosion limited to a single point
2	Mild to moderate erosion in a limited area
3	More extensive erosion
4	General damage.

Supplementary Figure

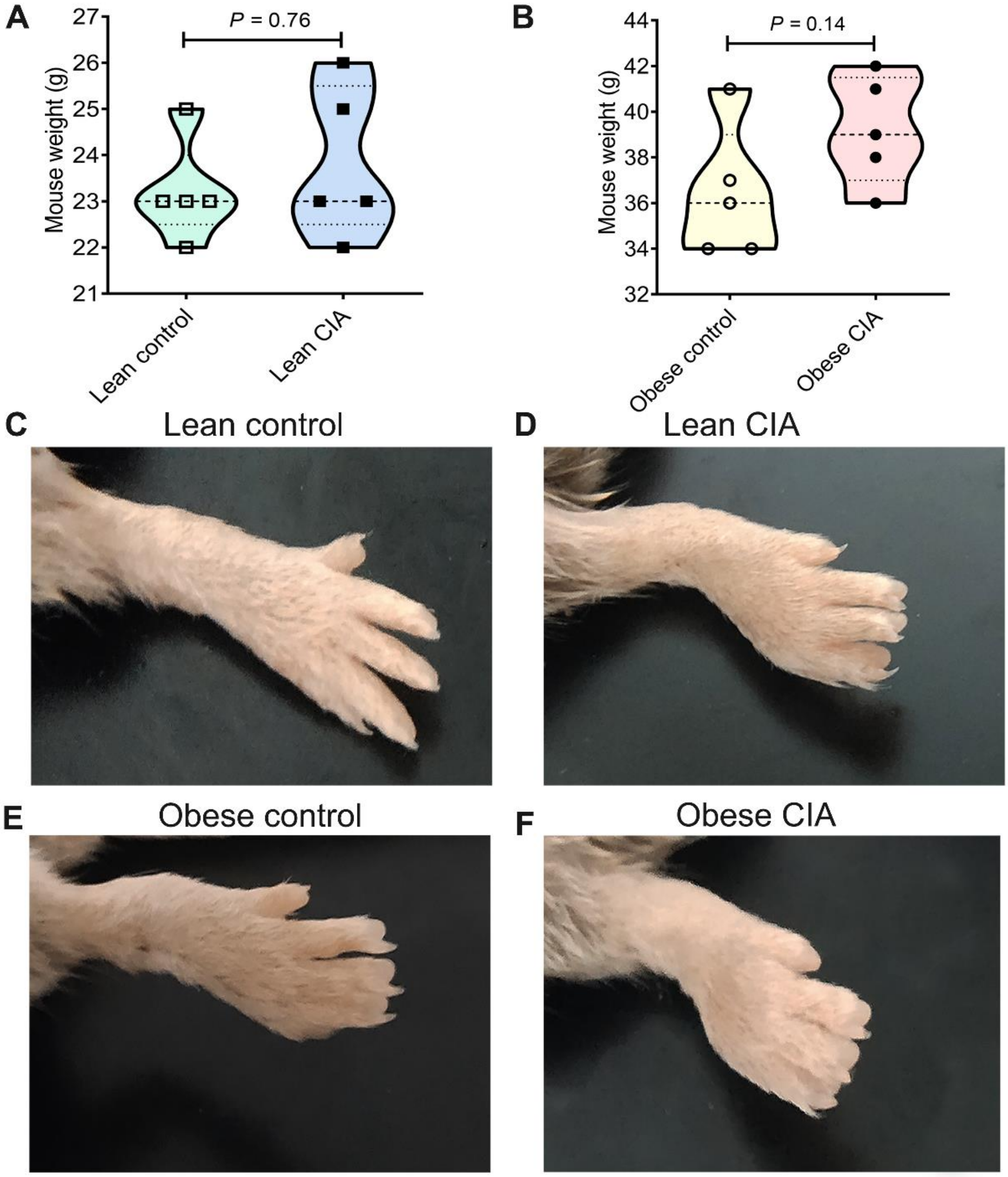

Figure S1. Baseline characteristics and arthritic phenotypes in Lean and Obese DBA/1 mice. (A-B) Body weight of mice at the time of the second randomization (immediately prior to CIA induction). Data are presented for each final experimental group (lean control, n=5; lean CIA, n=5; obese control, n=5; obese CIA, n=5). P values were calculated using the Mann-Whitney U test for comparisons within each diet cohort (lean control vs. lean CIA; obese control vs. obese CIA). Each symbol represents one mouse. (C-F) Representative photographs of hind paws from lean control and lean CIA (C, D) and obese control and obese CIA (E, F) mice at the study endpoint, illustrating the more severe arthritic swelling and joint enlargement in obese mice.

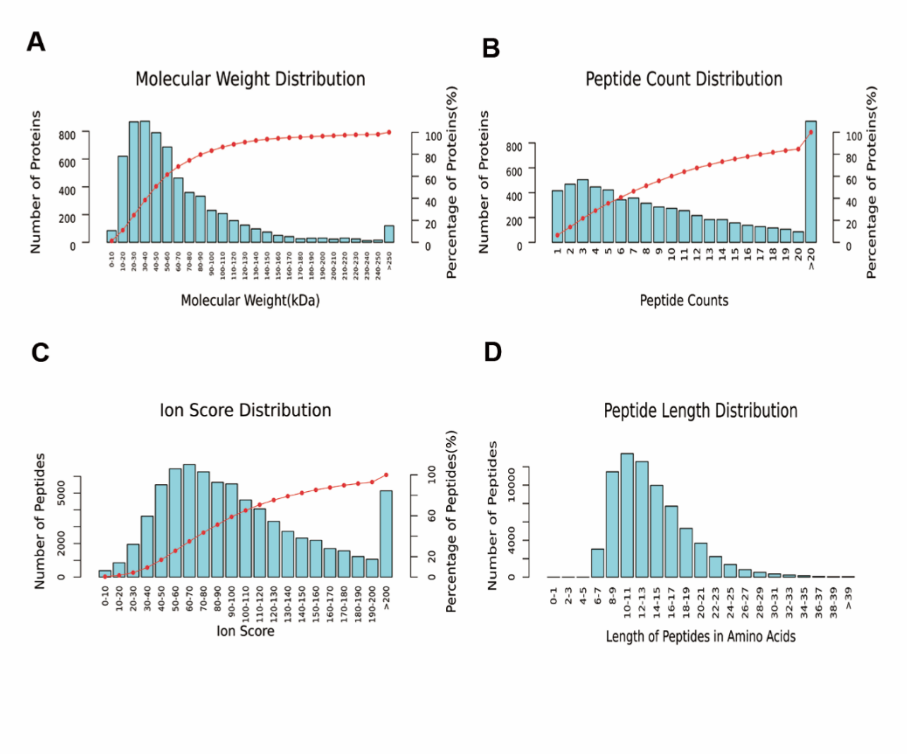

Figure S2. Evaluation of mass spectrometry identification results. (A-D) Graphs showing the distribution of peptide ion scores, protein relative molecular weight distribution, peptide sequence length distribution, and distribution of identified peptide quantity

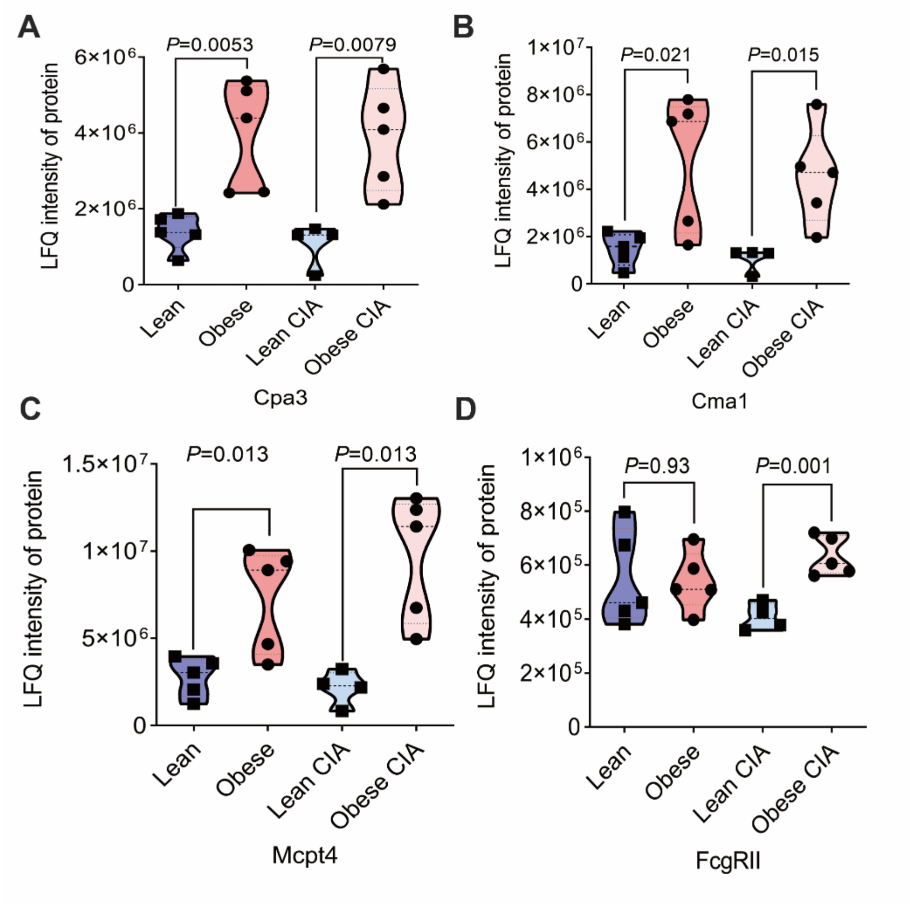

Figure S3. Differences in protein expression profiles of mast cell characteristic proteins in lymphocytes from lean and obese CIA mice. (A-D) Expression of CMA1, MCPT4, CPA3 and FcgRII were analyzed on total lymphocytes using proteomic data from lean and obese CIA mice with or without CIA treatment. *P* values calculated from Mann-Whitney U test. Each dot represents one animal.

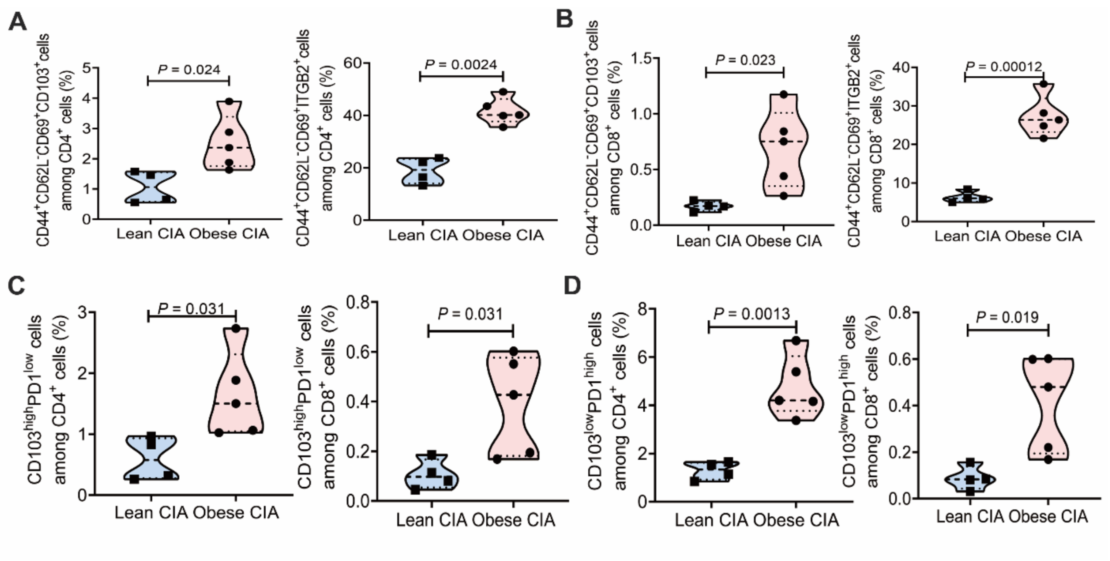

Figure S4. Differences in proportion of functional subgroups in TRM cells from lean and obese CIA mice. (**A-B**) Proportion of two subpopulation of resident memory T cells $CD103^{+}$ TRM and $ITGB2^{+}$TRM within $CD4^{+}$ or $CD8^{+}$T cells. (**C-D**) Proportion of two subpopulation of resident memory T cells Hobit enriched CD103 ^{high} PD1 ^{low} and Granzyme K-enriched CD103 ^{low} PD1 ^{high} TRM cells within $CD4^{+}$ or $CD8^{+}$T cells. P values calculated from Mann-Whitney U test. Each dot represents one animal.

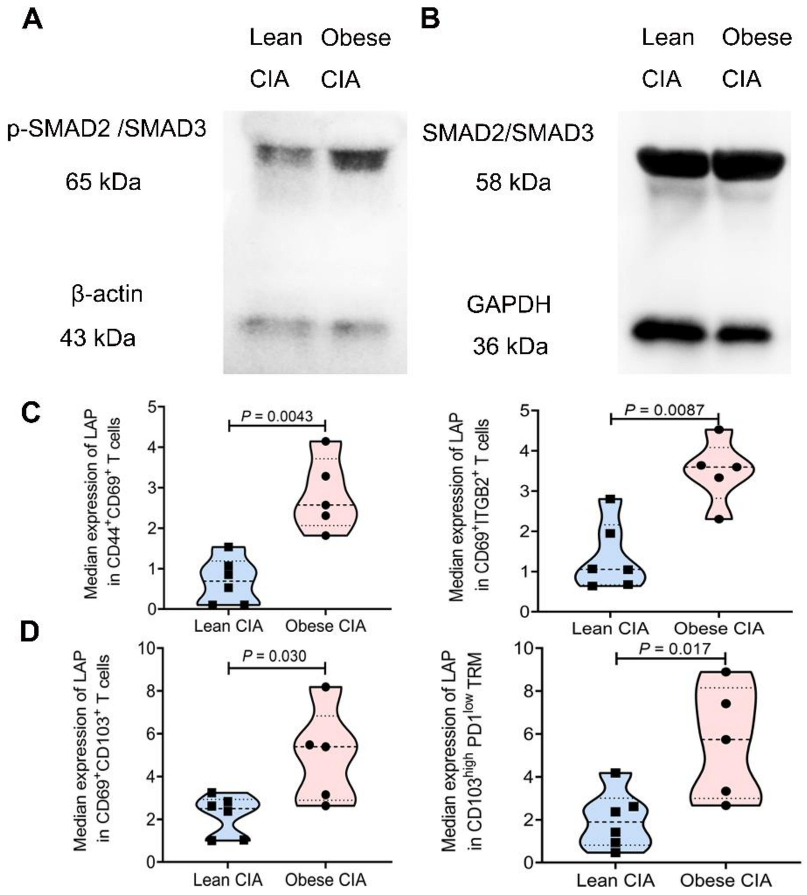

Figure S5. **Obesity modulates canonical TGF-β pathway activity.** (**A-B**) Lymph node cells from lean CIA and obese CIA mice were analyzed by Western blot for phosphorylated Smad2/3 (p-Smad2/3), total Smad2/3, GAPDH and β-actin (loading control). (**C-D**) Expression of LAP were analyzed on CD44^{+}CD69^{+} T cells, ITGB2^{+} TRM cells, CD103^{+} TRM cells specially CD103^{high}PD1^{low} TRM subsets in obese CIA mice compared to lean CIA mice. P values calculated from Mann-Whitney U test. Each dot represents one animal.

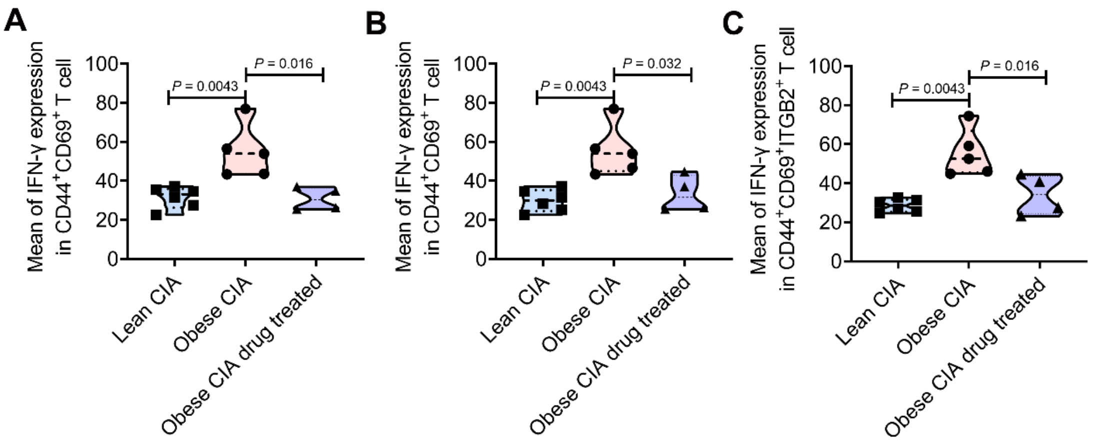

Figure S6. TGFβR signaling drives IFN-γ production in pathogenic TRM cells in obese CIA mice. (A-C) CyTOF analysis of normalized IFN-γ protein expression in T cell subsets. IFN-γ levels are significantly elevated in obese CIA mice compared with lean CIA mice. Asiaticoside treatment reduces IFN-γ expression in all three subsets in obese CIA mice ($P < 0.05$), indicating that TGFβR hyperactivation promotes a pro-inflammatory phenotype in these TRM-like and TRM cells. *P* values calculated from Mann-Whitney U test. Each dot represents one animal.

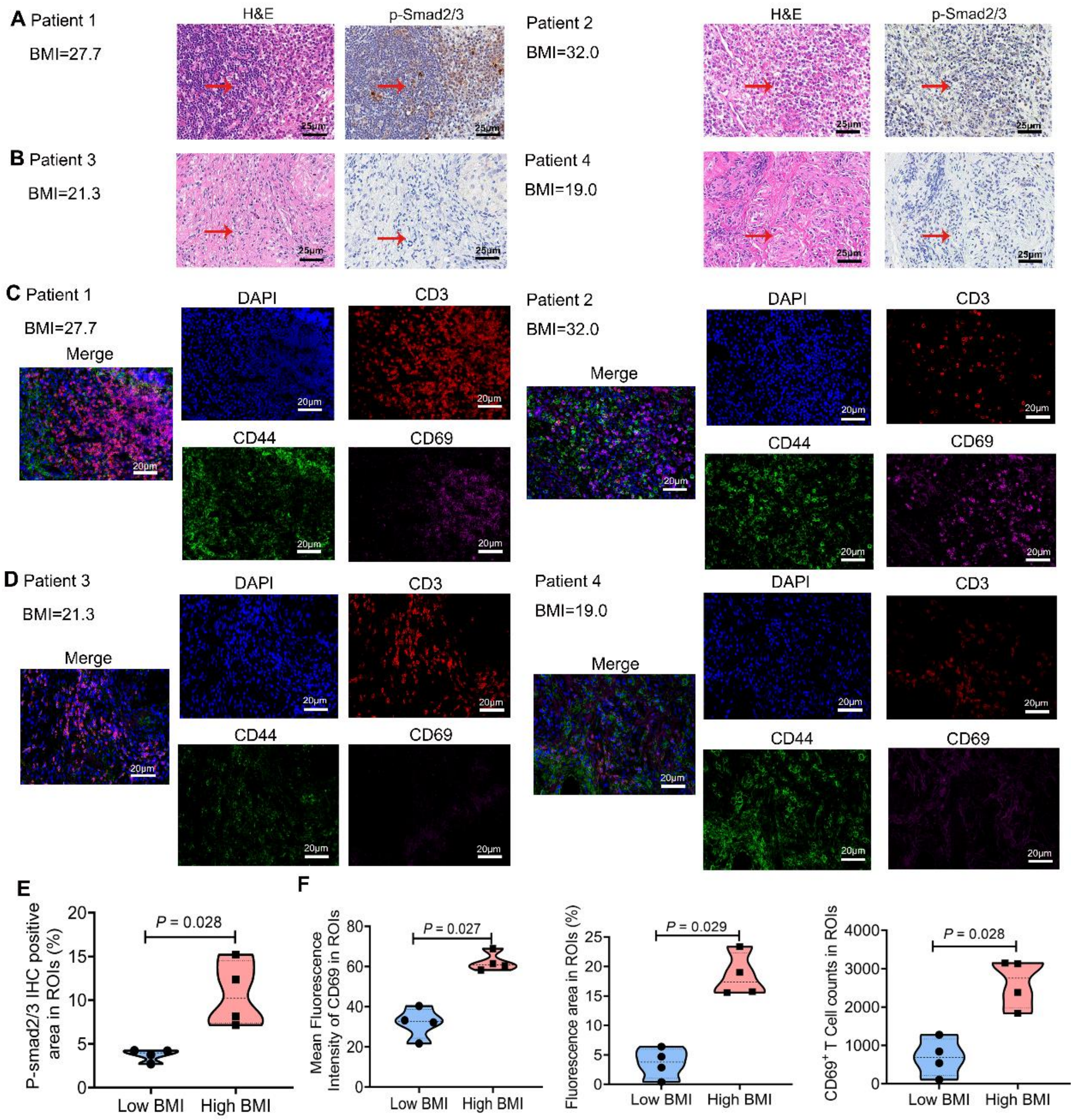

Figure S7. Preliminary evidence for synovial TRM-like cell accumulation and elevated TGFβ pathway activity in high-BMI RA patients. (A-B) Representative images of synovial tissues from high-BMI (Patient 1, BMI=27.7; Patient 2, BMI=32.0) and low-BMI (Patient 3, BMI=21.3; Patient 4, BMI=19.0) RA patients, showing H&E staining (left) and p-Smad2/3 immunohistochemistry(right) in adjacent sections. Scale bars:25 μm.(C-D) Representative multiplex immunofluorescence images of synovium stained for CD3 (red), CD44 (green), CD69 (cyan/white), and DAPI (blue) in (C) high-BMI patients and (D) low-BMI patients. Scale bars: 20 μm. (E) Quantification of p-Smad2/3 positive area in synovial ROIs. (F) Quantification of CD69 MFI, positive area, and density of $CD3^{+}CD44^{+}CD69^{+}$ TRM-like cells in synovial ROIs. Data points represent individual ROIs (n=4 per group, from two patients per group). P values were calculated using the Mann-Whitney U test.

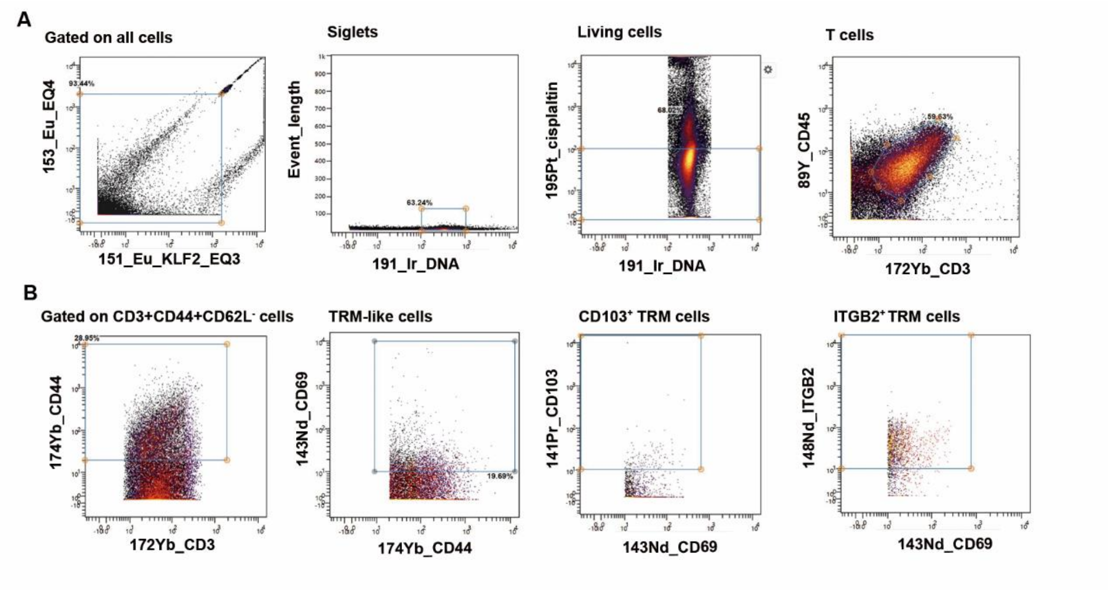

Figure S8. The gating strategy of TRM cells. A representative mass spectrometry flow cytometry diagram depicting the breakdown of lymphocytes using gating strategies in data analysis. (**A**) Determine individual cell populations based on Ir staining, select live cells with negative cisplatin staining, and then recognize $CD45^{+}CD3^{+}$T cells. **(B)** Effector and resident memory T cells are then determined by CD44 and CD69(CD62L results not shown, 99% negative) expression, followed by analysis of CD103 and ITGB2 expression in the rightmost panel.
